# Greater functional diversity and redundancy of coral endolithic microbiomes align with lower coral bleaching susceptibility

**DOI:** 10.1038/s41396-022-01283-y

**Published:** 2022-07-15

**Authors:** Anny Cárdenas, Jean-Baptiste Raina, Claudia Pogoreutz, Nils Rädecker, Jeremy Bougoure, Paul Guagliardo, Mathieu Pernice, Christian R. Voolstra

**Affiliations:** 1 Department of Biology, University of Konstanz, Konstanz, 78457 Germany; 2 Red Sea Research Center, Division of Biological and Environmental Science and Engineering, King Abdullah University of Science and Technology, Thuwal, 23955 Saudi Arabia; 3 Climate Change Cluster, University of Technology Sydney, Ultimo, NSW 2007 Australia; 4 Laboratory for Biological Geochemistry, School of Architecture, Civil and Environmental Engineering, École Polytechnique Fédérale de Lausanne, Lausanne, 1015 Switzerland; 5 Centre for Microscopy, Characterisation and Analysis, The University of Western Australia, Perth, WA 6009 Australia

**Keywords:** Microbial ecology, Microbial ecology

## Abstract

The skeleton of reef-building coral harbors diverse microbial communities that could compensate for metabolic deficiencies caused by the loss of algal endosymbionts, i.e., coral bleaching. However, it is unknown to what extent endolith taxonomic diversity and functional potential might contribute to thermal resilience. Here we exposed *Goniastrea edwardsi* and *Porites lutea*, two common reef‐building corals from the central Red Sea to a 17-day long heat stress. Using hyperspectral imaging, marker gene/metagenomic sequencing, and NanoSIMS, we characterized their endolithic microbiomes together with ^15^N and ^13^C assimilation of two skeletal compartments: the endolithic band directly below the coral tissue and the deep skeleton. The bleaching-resistant *G. edwardsi* was associated with endolithic microbiomes of greater functional diversity and redundancy that exhibited lower N and C assimilation than endoliths in the bleaching-sensitive *P. lutea*. We propose that the lower endolithic primary productivity in *G*. *edwardsi* can be attributed to the dominance of chemolithotrophs. Lower primary production within the skeleton may prevent unbalanced nutrient fluxes to coral tissues under heat stress, thereby preserving nutrient-limiting conditions characteristic of a stable coral-algal symbiosis. Our findings link coral endolithic microbiome structure and function to bleaching susceptibility, providing new avenues for understanding and eventually mitigating reef loss.

## Introduction

The health and ecological success of reef-building corals are supported by a wide range of symbiotic microorganisms, prompting scientists to consider this consortium as a discrete biological unit—the coral holobiont [1, 2]. The past thirty years of research have provided important insights into the diversity, biogeography, and functional roles of the microorganisms associated with corals. The most well-known coral symbionts are dinoflagellates from the Symbiodiniaceae family [3], which fulfill most corals’ energy requirements [4]. These microalgae are the photosynthetic engines of the reef, powering the metabolically expensive process of coral calcification [4]. In addition to Symbiodiniaceae, coral tissues also harbor an array of protists, fungi, bacteria, archaea, and viruses, which play numerous roles in coral health [5, 6]. These other microorganisms have been implicated in the cycling of essential nutrients [7–11], production of antimicrobial and antioxidant compounds [12–14], protection against UV radiation [15], and coral larval settlement [16, 17]. Although interspecies interactions occurring in the coral tissue and mucus are the focus of many studies, these compartments are usually less than 3 mm thick and, metaphorically speaking, represent the tip of the iceberg with respect to the volume of a coral [18, 19]. Indeed, the vast expanse of the underlying skeleton harbors an abundance of endolithic microorganisms, but the functional roles of these endoliths and their contribution to the health of the coral holobiont remain poorly understood [18, 19].

The most common photosynthetic organisms in the coral skeleton belong to a taxonomically diverse group of filamentous green algae from the genus *Ostreobium* [20, 21], which form dense bands below the tissue of many coral species [22]. Members of this genus can rapidly colonize the skeleton of coral juveniles following their settlement [23]. Under normal conditions, these photosynthetic algae receive very low light levels [24] and are well adapted to photosynthesize in near-darkness [25]. However, when corals bleach following environmental stress, the expulsion of the intracellular Symbiodiniaceae cells from coral tissues results in an increase in the amount of light reaching *Ostreobium*, enabling these endolithic algae to rapidly photo-acclimate and bloom [26–28]. Endoliths can directly interact with the coral tissue through the transfer of photosynthates and constitute a source of energy or nutrients for the host during environmental stress [29–31]. In addition, the prokaryotic communities inhabiting the coral skeleton are diverse and spatially structured, exhibiting high levels of intra-colony heterogeneity [32, 33]. These endoliths likely contribute to nitrogen, sulfur, and carbon cycling [34], and may be an important source of limiting nutrients for the overlying coral tissue [35]. Endolithic communities therefore potentially play an underacknowledged but fundamental role in the health of their coral hosts.

Unlike the microorganisms associated with coral tissues, endolithic communities seem less sensitive to specific environmental perturbations, such as ocean acidification [32]. However, with the exception of stimulated endolithic photosynthesis during coral bleaching, the response of the endolithic communities to heat stress in terms of community structure, functional potential, and nutrient cycling is currently unknown. Since 2015, more than 75% of reefs worldwide have been affected by mass bleaching events [36], and nearly half of the coral cover has been lost since the mid-80s on the largest reef system in the world, the iconic Great Barrier Reef [37, 38]. Given the escalating impact of marine heatwaves and rising sea surface temperature on coral reef assemblages [39], understanding the microbial processes underpinning coral health, including the overlooked endolithic communities, constitutes an important research priority.

Here we combine metagenomics with high-resolution chemical imaging (NanoSIMS) to characterize the metabolic capacity and putative contribution of endolithic microbes to coral holobiont functioning during heat stress. We compared the metabolic dynamics under heat stress of two species of massive reef-building corals, *Goniastrea edwardsi* and *Porites lutea*, which are abundant in the central Red Sea and common across the Indo-Pacific [40]. Both species are among the most environmentally resilient corals in the Indo-Pacific [41], and in particular, the genus *Porites* is regarded to have high thermal tolerance [42, 43]. These two species have different skeletal properties that likely affect the structure and functions of their endolithic communities with *G. edwardsi* exhibiting a denser and less porous skeleton compared to *P. lutea* [44]. We aimed to (*i*) taxonomically and functionally compare the endolithic communities of these two corals species; (*ii*) determine whether experimentally induced heat stress differentially impacts these communities taxonomically and functionally, in particular with regards to pathways involved in energy acquisition and cycling; and (*iii*) quantify the impact of heat stress on carbon and nitrogen assimilation by endolithic microbes.

## Methods

### Coral sampling & heat stress experiment

Corals were collected using SCUBA from a sheltered inshore reef off KAUST (Abu Shoosha reef: 22°18’16.3”N, 39°02’57.7”E, on 04 February 2019), from approximately 5–8 m of water depth. In total, six colonies of *P. lutea* and *G. edwardsi* were collected. All collected coral colonies showed prominent bands in their skeleton. Coral colonies were transferred to the Coastal and Marine Resources Core Lab (CMOR) at KAUST and fragmented directly after collection. Two fragments (one for each treatment: “control” and “heat stress”) were generated for each of the six colonies, resulting in a total of 24 fragments. As both collected coral species displayed massive growth forms, the skeletal areas that became exposed after fragmentation were sealed off with two-component epoxy putty to ensure that biotic and abiotic conditions of the skeleton were not altered by the direct contact with seawater or increases in light availability. All fragmented corals were maintained in a shared 100 L aquarium for a four-day acclimation phase and subsequently distributed into individual flow-through aquaria (100 L each; each aquarium containing one colony fragment of each species) just before the experiment. All aquaria were continuously supplied with sand-filtered seawater freshly collected from a near-shore reef and equipped with 600 W aquaria heaters (Schego, Offenbach, Germany) and Radion light systems (Ecotech Marine Inc., Bethlehem, PA). During the initial acclimation, seawater temperatures and light levels used during the experiment mimicked the ambient conditions recorded at nearby shallow reefs at the depth of coral collection: 27 °C and 750 µmol photons m^−2^ s^−1^ (at peak irradiation) in a 12:12 h light:dark cycle. After the initial acclimation phase, seawater temperature was ramped up to 33 °C in half of the aquaria over the course of 3 days (i.e., an incremental increase by 1 °C every 12 h; subsequently referred to as “heat stress” treatment), while the other half of the aquaria was maintained at a constant 27 °C (“control” treatment). Heat stress temperature corresponds to the absolute summer maximum temperature recorded in 2017 [45] and the control corresponds to the measured in situ temperature when corals were collected. The temperature treatments were maintained until *Porites* sp. fragments were visibly bleached at 33 °C, which occurred after 17 days (14 days at maximum temperature). Of note, *G. edwardsi* did not exhibit any signs of bleaching at that time.

### Stable isotope labelling

On day 17 of the experiment, corals were collected from both temperature treatments and subjected to an incubation period of 24 h in 330 ml beakers filled with isotope-enriched artificial seawater (*n* = 6 per species and treatment). Artificial seawater composition (salinity = 39 PSU, pH = 8.1, 492.5 mM NaCl, 46.23 mM MgCl_2_, 10.8 mM Na_2_SO_4_, 9.0 mM CaCl_2_, 7.9 mM KCl) was adapted after Harrison et al. [46] to mimic Red Sea conditions and supplemented with 3 mM ^13^C-bicarbonate (H^13^CO_3_^−^) and 5 mM ^15^N-ammonium (^15^NH_4_^+^) for the isotope labeling incubations. During the incubation, corals from heat stress and control treatments were kept in temperature-controlled water baths equipped with Radion light systems (Ecotech Marine Inc., Bethlehem, PA) to maintain temperature and light regimes according to treatment conditions. Throughout the incubation, the seawater in the beakers was constantly stirred at 300 rpm using magnetic stirring bars to homogenously mix the water volume. For each coral species, one fragment was placed in artificial seawater without any isotopic label (isotope label replaced with unlabeled counterparts) to act as control for subsequent NanoSIMS analyses.

### Coral sectioning/sampling for next-generation sequencing and NanoSIMS

After the end of the 24 h labelling incubations, coral fragments were collected and immediately sectioned into slices using an electric rotating diamond saw blade. Separate slices from each fragment were used for (a) Next-Generation Sequencing (NGS), (b) NanoSIMS analyses, and (c) spectral imaging of endolithic bands (*n* = 6 for each application). Sections were kept on ice until further processing. Using a Dremel 4000 rotary tool (Dremel, Prospect, IL), individual sections were trimmed for subsequent sampling. For nucleotide extractions, individual fragments were cut from each slice from (a) coral tissue (defined here as “Tissue”), (b) visible green or pink endolithic bands (defined here as “endolithic band”), and (c) white skeleton without any visible endolithic colonization (defined here as “skeleton”). For NanoSIMS imaging, one fragment per sample (approximately 15 × 5 × 2 mm) containing the coral tissue as well as the underlying skeleton and endolithic communities was collected. Between individual coral fragments and sections, the milling blade was thoroughly rinsed with 70% ethanol to avoid carry-over of tissue and/or microbes between sections. Sections for NGS sequencing were immediately ground manually to a fine powder over liquid nitrogen using a cryogrinder mortar and pestle. Powdered samples were transferred into 2 ml Eppendorf tubes, snap-frozen in liquid nitrogen, and stored at −80 °C until further processing. Sections for NanoSIMS were immediately transferred to fixation buffer (2.5% glutaraldehyde and 1% formaldehyde in PBS-sucrose buffer, pH 7.5) and stored for 24 h at 4 °C.

### Photographic documentation and hyperspectral imaging analysis

For each coral sample, one slice was used for conventional photography and hyperspectral imaging analysis. For spectral imaging, sections were illuminated under constant light by two full-spectrum lights (Specim IQ accessory pro kit). Hyperspectral images were recorded with a tripod-mounted Specim IQ camera (Specim, Spectral Imaging LTD, Oulu, Finland) following calibration using the provided white reference tile. Recorded representative spectra were extracted from the coral tissue, the endolithic band, and the underlying coral skeleton for each of the sections using the IQ studio software. Selected spectral regions were statistically compared based on the distance between their cumulative distributions using a Kolmogorov-Smirnov (KS) test (which provides a measure of dissimilarity between the distributions).

### DNA extraction and sequencing library preparation

Between 50–100 mg of each powdered sample was weighed directly into a clean 1.5 ml Eppendorf tube. Initial lysis of samples was conducted in 360 µl of ATL buffer + 20 µl of Proteinase K (20 mg/ml) per reaction by incubation at 56 °C and 900 rpm overnight. DNA extractions were performed using the Qiagen QIAamp Investigator Kit (Qiagen, Hilden, Germany) following manufacturer’s instructions for DNA extraction from bone. In the final elution step, DNA was eluted from Qiagen membranes in 25 µl of ATE buffer and stored at −20 °C until further processing. Negative controls (i.e., no template DNA PCR and no sample DNA extraction) were performed to account for contaminants in bacterial community analysis. Quantification of DNA was conducted using a Qubit 2-Fluorometer (Invitrogen, Waltham, MA).

### Amplicon and metagenomic library preparation

Amplicon libraries were prepared using the Nextera XT Index Kit (Illumina, San Diego, CA) according to manufacturer’s instructions (Illumina). In brief, each 10 µl PCR reaction contained the Qiagen multiplex PCR kit (Qiagen, Hilden, Germany), 10 ng of DNA, and primers at a final concentration of 0.3 μM. Cycling conditions were 95 °C for 15 min, followed by 27 cycles of 95 °C for 30 sec, 55 °C for 90 sec, 72 °C for 30 sec, with a final extension of 72 °C for 10 min. The hyper-variable region V5-V6 of the bacterial 16 S rRNA gene was amplified using the primers 784 F (5′-TCGTCGGCAGCGTCAGATGTGTATAAGAGACAGAGGATTAGATACCCTGGTA-3′) and 1061R (5′-GTCTCGTGGGCTCGGAGATGTGTATAAGAGACAGCRRCACGAGCTGACGAC- 3′; Illumina overhang adaptor sequences are underlined) [47, 48]. Samples were amplified in triplicates which were combined before cleaning with ExoProStar 1-step (GE Healthcare, Pollards Wood, UK), indexed for 8 cycles, and confirmed on a 1% agarose gel. Samples were normalized using the SequalPrep Normalization Plate Kit (Invitrogen, Waltham, MA). Equimolar ratios of each sample were pooled and quantified using the Qubit dsDNA HS assay with the Qubit 2.0 Fluorometer (Thermo Fisher Scientific, Waltham, USA). Pooled libraries were re-quantified using the qPCR KAPA Biosystems library quantification on an ABI HT7900 system (Applied Biosystems, Waltham, MA) and sequenced on the MiSeq platform (Illumina, San Diego, US) with 20% PhiX using the 2 × 301 bp paired-end V3 chemistry at the KAUST BioScience Core Lab (BCL) sequencing facility.

An average of 160 ng of genomic DNA per sample were used as an input to prepare metagenomic libraries using the Ovation Ultralow Library System V2 (Tecan, Männedorf, Switzerland). Briefly, genomic DNA was sheared to fragment sizes of approximately 250–300 bp using the Covaris M2 (90 s, 20 °C, 20% duty factor). Fragments were end-repaired, bidirectionally ligated to sequencing adaptors, and amplified with adaptor-specific primers using 8 PCR cycles. The quality of the libraries was checked on a Bioanalyzer DNA 1000 Chip (Agilent Technologies, Santa Clara, CA), followed by quantification using the Qubit HS DNA system (Invitrogen, Waltham, MA). Libraries were pooled using equimolar ratios to a final concentration of 10 nM. Pooled libraries were re-quantified using the qPCR KAPA Biosystems library quantification on an ABI HT7900 system (Applied Biosystems, Waltham, MA) and paired-end sequenced (2 × 151 bp) on the HiSeq 4000 (Illumina, San Diego, US) platform at 1.8 pM with 1% PhiX at the KAUST BCL sequencing facility.

### Sequencing data analysis

#### 16S rRNA gene amplicon sequencing

Amplicon sequence variants (ASVs) were inferred using DADA2 v1.21.0 [49]. In brief, forward and reverse sequence reads were truncated at the 3′ end at 290 and 210 base pairs, respectively. Reads with expected errors >2 or with the presence of ambiguous bases were discarded. A denoising step was done using the “pool=T” option to increase the resolution of singletons and doubletons. ASVs inferred from individual read pairs were merged and checked for chimeras. Taxonomic annotation was done using the SILVA database (version 138) [50]. Sequence reads statistics are found in Table S1 and ASV raw counts are found in Table S2. Relative abundances of the most abundant 10 prokaryotic families were generated using ggplot2 v3.3.5 [51]. PCA analyses were calculated from Euclidean distances of centered-log-ratio (clr)-transformed ASV counts using phyloseq v1.34.0 [52], and differences between bacterial communities were tested using PERMANOVA as implemented in the R package Vegan v2.5 [53]. For alpha diversity estimation, ASV counts were aggregated to families, and the number of observed families was calculated based on rarefied abundances to a sample count of 1966. The identification of differentially abundant ASVs was done using the Analysis of Composition of Microbiomes with Bias Correction (ANCOM-BC) function [54]. ANCOM-BC v1.0.5 function was applied on ASVs absolute counts to compare species and skeletal compartments samples using the False Discovery Rate (FDR) method to correct for multiple testing. A feature (i.e., ASV) was considered enriched in a specific condition when it was significantly more abundant than the reference (i.e., “control”) based on the ANCOM-BC analysis (i.e., FDR-adjusted *p* values were <0.05).

#### Gene-centric metagenomic analysis

Sequencing reads were adapter- and quality- (bases with quality scores <20) trimmed using Trimmomatic v.0.38 [55]; reads shorter than 50 nt were removed from the analysis. Assemblies produced by MEGAHIT v1.1.1-2 [56], metaSPAdes v3.13.0 [57], and IDBA v1.1.3-1 [58] were evaluated using QUAST v5.0.2 [59]. The best assembly was produced by MEGAHIT (Table S4) using K-mer lengths of 141. Open reading frames (ORF) were identified using Prodigal v2.6.3 [60], and total read counts to each ORF were estimated using Salmon v0.7.2 [61]. Contigs shorter than 500 bp were excluded from downstream analysis. Taxonomic annotation was done using Kaiju v1.7.3 [62] using the minimum exact matches (MEM) mode with a word length of 11 against the NCBI nr database that includes archaea, bacteria, viruses, and microbial eukaryotes. Functional annotation of ORFs against the KEGG database was done using KOfamscan v 1.3.0 [63], and the best match per contig was chosen based on the lowest E-value (considering only E-values <0.001) and highest bit score (considering only scores >100). ORFs were aggregated into KOs and L3 pathways by adding counts from contigs annotated to the same KO/L3 pathways. A total of 15,213 KOs affiliated to 457 KEGG L3 pathways were annotated, but data analysis and inferences were only made with pathways annotated to metabolism (KEGG map ko00001, 3975 annotated KOs and 160 L3 pathways). Family and metabolism KO alpha diversity was estimated by the Shannon index [64] on rarefied abundances to a sample count of 698,219 and 686,534, respectively, using Vegan [53]. Generalized linear mixed models were fit to test for differences in alpha diversity between species, treatments, and compartments, accounting for a random effect of the genotype using the *lmer* function of the lme4 v3.1 package [65]. Differentially abundant KOs, L3 pathways, and taxonomic families between coral species and treatments were identified using ANCOM-BC [54]. A feature was considered enriched in a specific condition when it was significantly more abundant than the reference (i.e., “control”) based on the ANCOM-BC analysis (i.e., FDR-adjusted *p* values <0.05). Ordination plots were computed from Euclidean distances of clr-transformed counts and represented in principal component analysis (PCAs) to account for the compositionality of the data according to Aitchison’s methodology [66]. Functional signatures that best predict species and skeletal compartments were identified using the Selbal algorithm v0.1 based on compositional balances (i.e., ratios of relative abundances) [67]. Selbal was run on KEGG L3 pathways involved in C metabolism, and the predictions were tested by a cross-validation procedure.

#### Genome-centric metagenomics analysis

In addition to the gene-centric metagenomic analysis, we did metagenomic binning by mapping trimmed reads to the best assembly using Bowtie2 v2.4.1 [68]. Mapping files were used as input files for Metabat2 2.11.1 [69], CONCOCT2 [70], and maxbin2 [71] to calculate tetranucleotide frequencies and differential contig coverage. The resulting bins were evaluated using checkM v1.1.2 [72], and the best binning results among the three software were chosen using MetaWRAP v1.2.1 [73]. Bins with completeness >75% and contamination <10% were considered metagenome-assembled genomes (MAGs). In addition, bins were dereplicated at 95% average nucleotide identity (ANI) using dRep v3.2.2 [74]. Taxonomic placement of MAGs was done based on ANI with the “classify_wf” workflow of GTDB-tk v1.3.0 [75] using the Genome Taxonomy Database (GTDB) release 95 [76]. Functional annotation was done using KOfamscan v 1.3.0 [63], Prokka v1.13 [77], and METABOLIC v2.0 [78]. Completeness of KEGG modules was evaluated using EnrichM v0.4.3 (available at: https://github.com/geronimp/enrichM). Phylogenomic analysis was done using the UBCG pipeline v0.1 [79]. Differential abundance analysis was done using ANCOM-BC using clr-transformed mapped reads to each MAG, results were represented on heatmaps indicating differentially abundant MAGs between species.

### Sample embedding and preparation for SEM and NanoSIMS

Coral cross sections for NanoSIMS were preserved in fixation buffer (2.5% glutaraldehyde and 1% formaldehyde in PBS-sucrose buffer, pH 7.5) for 24 h at 4 °C. After the fixation, samples were transferred to 3× PBS-sucrose buffer at 4 °C until embedding.

Initially, the entire coral cross sections (*n* = 6 per treatment) were embedded into resin. Coral cross sections were dehydrated with increasing concentrations of ethanol (50%, 70%, 90%, 100%), followed by acetone (100%), and then infiltrated with increasing concentrations of Araldite 502 resin (20%, 30%, 40%, 50%, 60%, 75%, 100%; Sigma-Aldrich) under vacuum over a total of 48 h. Coral cross sections were then finally transferred into 1-inch circular molds (3 biological replicates were added to each mold; Figure S1A-B), covered with 5 mm of Araldite 502 resin (100%), and cured at 60 °C under vacuum for 24 hours. The resin-encased coral cross sections were polished down to 1 µm using a RotoPol-35 (Struers, Denmark). Resin blocks were then coated with 5 nm of gold, mounted on SEM specimen holders and mapped using a Zeiss EVO Scanning Electron Microscope (Zeiss, Germany). For each sample, three areas (120 × 80 µm) from each microenvironment (coral tissue, endolithic band, and skeleton) were imaged in high resolution with SEM to be targeted with NanoSIMS imaging. Following SEM, resin blocks were then transferred to a 1-inch sample holder for NanoSIMS analysis.

### NanoSIMS analysis

The NanoSIMS-50 (Cameca, France) at the Centre for Microscopy, Characterisation and Analysis (CMCA) at the University of Western Australia was used for all subsequent analyses. The NanoSIMS-50 allows simultaneous collection and counting of five isotopic species, which enables the determination of ^13^C/^12^C and ^15^N/^14^N ratios. Enrichments of the rare isotopes ^13^C and ^15^N were confirmed by an increase in their isotopic ratios above natural abundance values recorded in controls (^13^C/^12^C: 0.0110 ± 0.0001 and ^15^N/^14^N: 0.0037 ± 0.0001, *n* = 190 cells). NanoSIMS analysis was undertaken by rastering a 2.5 pA Cs^+^ beam (~100 nm diameter) across defined 35 µm^2^ sample areas (256×256 pixels) with a dwell time of 30 ms per pixel. The isotope ratio values are represented hereafter using a colour-coded transform (hue saturation intensity (HSI)) showing natural abundance levels in blue and grading to high enrichment in pink. Images were processed and analyzed using Fiji (http://fiji.sc/Fiji) with the Open-MIMS plug-in (http://nrims.harvard.edu/software). All images were dead-time corrected. Quantitative data were extracted from the mass images through manually drawn regions of interest.

Given that our initial embedding of full coral sections did not yield usable NanoSIMS data (see Results; Figure S1H-O), we used an alternative method to prepare the samples for NanoSIMS. Specifically, fixed coral cross sections were further sub-divided into tissue and endolithic band using a 38 mm diamond cutting wheel and decalcified in 0.5 M EDTA solution over 10 days (solution was changed once after 5 days). Decalcified samples were post-fixed in 1% OsO_4_ (1× in PBS, final pH 7.5) for 2 h and dehydrated with increasing concentrations of ethanol (50%, 70%, 90%, 100%), followed by acetone (100%). Following this procedure, samples were infiltrated with increasing concentrations of SPURR resin (25%, 50%, 75%, 100%) and cured at 65 °C overnight. Finally, samples were sectioned (200 nm) using an ultramicrotome (Leica Microsystems), mounted on silicon wafers, and coated with 5 nm of gold (for NanoSIMS analysis). For electron microscopy, 100 nm thick sections were mounted onto copper TEM finder grids (coated in formvar—Emgrid, Australia).

## Results

**Fig. 1:**
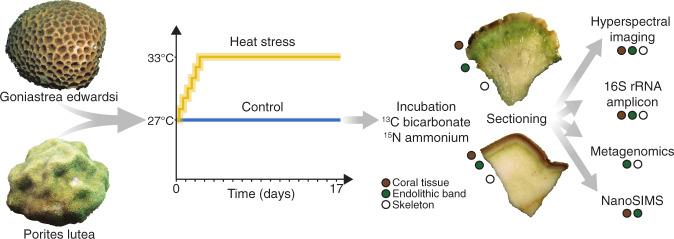
Experimental setup. Coral fragments from *G. edwardsi* and *P. lutea* colonies (*n* = 6) were subjected to a heat stress experiment followed by a 24 h incubation with stable isotope tracers before sample collection and sectioning. Subsequently, we measured the hyperspectral profile of each section and conducted 16S rRNA gene amplicon sequencing, metagenomics, and NanoSIMS analyses to characterize the endolithic microbial communities and their associated metabolic potential as well as differential ^13^C and ^15^N assimilation under heat stress.

To assess the putative functional roles of endolithic microbiomes in coral bleaching, we characterized their taxonomic and metabolic diversity, together with measurements of N and C assimilation during heat stress. For this, we exposed fragments of *G. edwardsi* and *P. lutea* to a 17-day heat-stress experiment and combined hyperspectral imaging, 16S rRNA gene amplicon sequencing, metagenomics, and NanoSIMS imaging on different skeletal compartments (Fig. 1).

### *G. edwardsi* and *P. lutea* have heterogenous skeletal banding patterns

**Fig. 2:**
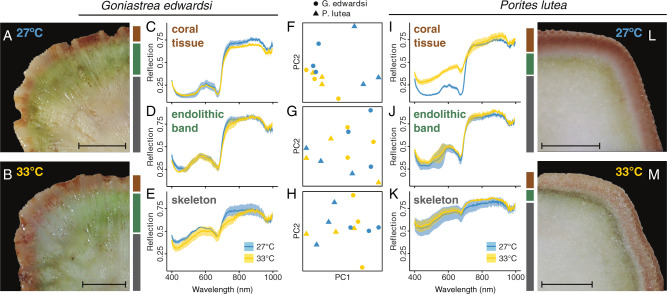
Hyperspectral imaging of coral fragments from *G. edwardsi* and *P. lutea* under heat stress. Representative picture of skeleton banding patterns of a *G. edwardsi* colony at **A** control (27 °C), **B** heat-stress (33 °C), and hyperspectral profiles of **C** tissue, **D** endolithic band, and **E** skeleton. Hyperspectral profiles of *P. lutea* **I** tissue, **J** endolithic band, and **K** skeleton. Exemplary picture of skeleton banding patterns of a *P. lutea* colony at **L** control (27 °C) and **M** heat-stress (33 °C). Principal component analysis of the hyperspectral profiles of the **F **coral tissue, **G** endolithic band and **H** skeleton under control (27 °C) and heat stress conditions (33 °C). Means over all samples ± 95% CI. Scale bar: 1 cm.

Banding patterns visibly differed in their thickness and position (i.e., distance from tissues) between coral species but were also heterogeneous among fragments of the same coral species and often within the same colony (Figs. 2A, B, 2L, M, and S2). We sampled both the conspicuous (green or pink) endolithic band (found between 0 and 25 mm beneath the tissue in *G. edwardsi*, and 0 and 10 mm beneath the tissue in *P. lutea*) and the deep white skeleton underneath (with no visible endolithic colonization, approximately sampled 10 mm beneath the endolithic band). In addition, conspicuous pink bands were observed in two replicate colonies of *P*. *lutea*.

Hyperspectral profiles were indistinguishable between the tissues of *G. edwardsi* and *P. lutea* (Fig. 2C–K and S3). For all three compartments (i.e., coral tissue, endolithic band, skeleton), reflectance spectra followed patterns expected for chlorophyll-containing organisms, including reflectance minima in the blue and red spectra, higher reflectance in the green to orange domain, and a substantial increase in reflectance between 680 to 720 nm (commonly referred to as the red edge) followed by a plateau in the near-infrared domain [80] (Fig. 2C–E and 2I–K). However, some differences were apparent in the endolithic bands between the two species: *P. lutea*’s reflectance was significantly higher in the red edge (650 to 700 nm) and infrared (900–1000 nm) (KS test, *p* < 0.01; Figure S3). In addition, the reflectance of the skeleton was significantly higher in *P. lutea* compared to *G. edwardsi* across the entire 400 to 1000 nm range (KS test, *p* < 0.01), with a less pronounced red edge in *P. lutea* indicative of reduced chlorophyll concentrations.

After 17 days of heat stress, *P. lutea* fragments were visibly bleached, while *G. edwardsi* showed no evidence of symbiont loss (Figs. 2A, B and 2L, M). In congruence with the bleaching phenotype, hyperspectral imaging revealed contrasting optical properties between treatments in the tissues of *P. lutea*, in which heat-treated samples had a substantial and significant increase in reflectance in the 400 to 700 nm range and a less pronounced red edge (KS test, *p* < 0.01, Figs. 2C and 2I) likely reflecting the loss of chlorophyll-containing Symbiodiniaceae cells. No differences between heat-treated and control samples were observed in the skeleton or endolithic band spectra in *P. lutea* (Fig. 2J, K). However, reflectance significantly declined during heat stress in the skeleton of *G. edwardsi* in 400–500 and 700–800 nm ranges (KS test, *p* < 0.01, Fig. 2E). While the underlying causes of this shift are unknown, a similar decrease in the reflection in the 700–800 nm range has been previously described in plants during fungal pathogen infections [80, 81].

### Endolithic microbiomes of *G. edwardsi* are taxonomically more diverse

**Fig. 3:**
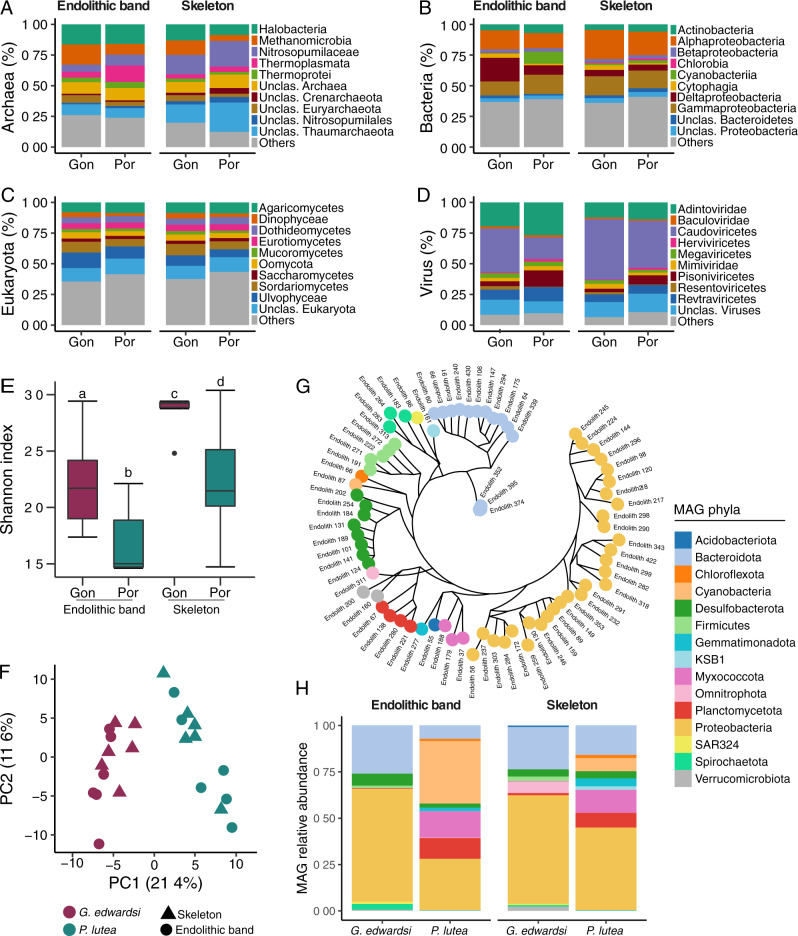
Taxonomic diversity of *G. edwardsi* and *P. lutea* metagenomes. Bar plots represent the relative abundance of the 10 most abundant taxonomic classes of **A** Archaea, **B** Bacteria, **C** Microbial eukaryotes, and **D** viruses in *G. edwardsi* (Gon) and *P. lutea* (Por). When annotation did not reach the class level, the highest taxonomic rank annotated was used. Less abundant classes were aggregated to the “Others” category. **E** Shannon diversity of all taxonomic families found in metagenomes. Significant differences in alpha diversity between species and skeletal compartments are denoted by letters above the boxes (Table S6). **F** Ordination plots of microbial composition at the family level in *G. edwardsi* and *P. lutea*. **G** Maximum likelihood phylogenetical inference of MAGs recovered from *G. edwardsi* and *P. lutea* metagenomes. A set of 92 house-keeping genes [79] were used for the reconstruction. **H** Relative abundance of MAGs clustered to the phylum level with respect to the total binned fraction. Sections **G** and **H** share color coding.

We used a metagenomic gene-centric approach (i.e., based on abundances of individual genes) to quantitatively compare the microbial taxonomic and functional diversity in the endolithic band and skeleton of the two coral species under ambient temperature. In both species, metagenomic sequences derived from the endolithic bands and skeletons were mostly of bacterial origin (~75%), followed by microbial eukaryotes (~5%), archaea (<1%), and viruses (<1%) (Figure S4). The unclassified fraction represented ~20% of the community, mainly derived from the coral host. At higher taxonomic ranks (Phylum/Class), taxonomic composition between coral species and skeletal compartments was overall similar (Fig. 3A–D), but significant differences existed at the family level between the endolithic band and the skeleton communities between both coral species (PERMANOVA, *p*_adj_ < 0.05, Fig. 3F, S5A–D, and S6). However, no significant differences were determined between skeletal compartments of the same coral species (Fig. 3F and Table S5). Family alpha diversity was also significantly higher in *G. edwardsi* than in *P. lutea* in both compartments (lmer, *p*_adj_ < 0.05), and the skeleton was more diverse than the endolithic band in both species (lmer, *p*_adj_ < 0.01, Fig. 3E and Table S6).

As the bacterial fraction represented most of the metagenomic reads, we further assessed bacterial diversity by 16S rRNA gene amplicon sequencing of tissue, endolithic band, and skeleton. The 16S rRNA analysis showed that alpha diversity at the family level consistently increased from tissue to endolithic band to skeleton (Figure S7C) and confirmed that significant differences exist in the composition of bacterial families between tissues and skeletal compartments (Figure S7A and Table S3). Nevertheless, some of the main features of bacterial diversity that were identified by metagenomics were overlooked by 16S rRNA gene sequencing (Fig. 3B, S7B, and S7D, E). A total of 6,491 ASVs assigned to 220 bacterial families were present in the 16S rRNA gene amplicon dataset, in comparison to 492 bacterial families identified through the gene-centric metagenomics approach (Figure S7D). Such discrepancies are common and, in the particular case of our study, may be due to the limited coverage of specific bacterial taxa by the 16S rRNA gene amplicon primers, but other factors (e.g., 16S rRNA secondary structures, gene copy number, coverage in databases) can also play a role [82, 83]. Despite the higher number of bacterial families identified using metagenomics, the most abundant bacterial families were consistent between metagenomes and amplicon data, and included members of the Desulfobacteraceae and Rhodobacteraceae (Figures S5B and S7B). In addition to bacteria, archaea, microbial eukaryotes, and viruses were also present and diverse in endolithic communities found in the metagenomic dataset. Specifically, we identified a total of: (i) 48 archaeal families, dominated by members of the classes Halobacteria, Methanomicrobia, and Thermoplasmata, in both coral species and skeletal compartments (Fig. 3A and S5A); (ii) 643 families of microbial eukaryotes, in which Ostreobiaceae (Ulvophyceae) and Symbiodiniaceae (Dinophyceae) were the two most abundant families in both species and compartments, representing on average 12.5% and 10.6% of the total microbial eukaryotic diversity of *G. edwardsi* and *P. lutea*, respectively (Fig. 3C, S5C). Notably, Fungi represented almost 50% of the microbial eukaryotic community and were mainly represented by a large diversity of Ascomycota (from the classes Sordariomycetes, Dothideomycetes, Eurotiomycetes and Saccharomycetes), Basidiomycota (class Agaricomycetes), and Mucoromycota (class Mucoromycetes) (Fig. 3C). Other low-abundant members of the microbial eukaryotic community belonged to the phyla Apicomplexa, Euglenozoa, and Chytridiomycota; (iii) 124 viral families, with the 10 most abundant families representing over 75% of the total viral community and mostly include members of the Caudovirales class such as *Myoviridae*, *Podoviridae*, and *Siphoviridae* (Fig. 3D, S5D).

Overall, 639 families (48.9%) belonging to bacteria, archaea, micro-eukaryotes, and viruses were differentially abundant between coral species in the endolithic band and 444 (34%) in the skeleton. In both compartments, more families were enriched in *G. edwardsi* in comparison to *P. lutea* (326 versus 313 in the endolithic band and 263 versus 181 in the skeleton; Figure S8 and Table S7). Most differences between coral species were explained by an enrichment in bacterial families of the Desulfobacterota (formerly belonging to the class of Deltaproteobacteria), including Desulfobacteraceae, Desulfohalobiaceae, and Desulfonatronaceae in the endolithic band and skeleton of *G. edwardsi*, while the skeletal compartments of *P. lutea* were enriched with families of the phylum Cyanobacteria, including Cyanobacteriaceae, Prochloraceae, and Microcoleaceae (Figure S9). The differential abundance analysis also revealed that Desulfurococcaceae and Ferroplasmaceae (Archaea), *Phycodnaviridae* and *Picobirnaviridae* (Viruses), and several families of the Sordariomycetes (Fungi) were enriched in *G. edwardsi* in comparison to *P. lutea*, while Cuniculiplasmataceae and Acidilobaceae (Archaea), *Secoviridae* and *Mimiviridae* (Viruses), and several members of the class Dothideomycetes (Eukaryota) were enriched in *P. lutea* in comparison to *G. edwardsi* (Table S7, Figure S8).

In addition to the gene-centric approach, we used a genome-centric approach to characterize the metabolic potential of bacterial populations present in the endolithic band and deep skeleton. We recovered a total of 75 bacterial Metagenome-Assembled Genomes (MAGs; completeness >75% and contamination <10%) from the skeletal compartments of *G. edwardsi* and *P. lutea* [84] that represented in average 6.91% of the metagenomes across samples (Figure S10 and Table S8). Differential abundance analysis identified 30 MAGs enriched in *G. edwardsi* (15 only in the endolithic band and 15 in the endolithic band and skeleton) and 21 MAGs enriched in *P. lutea* (8 only in the skeleton and 13 in the skeleton and endolithic band, Figure S11 and Table S8). The majority of MAGs were affiliated to the phylum Proteobacteria (Fig. 3G) and represented the most abundant fraction of the binned community (Fig. 3H). Besides members of the Proteobacteria, MAGs enriched in *G. edwardsi* were mainly affiliated to the phyla Desulfobacterota, Bacterioidota, and Firmicutes, while MAGs enriched in *P. lutea* were mainly affiliated to the phyla Cyanobacteria, Myxococcota, and Planctomycetota (Fig. 3H and S11).

Taken together, our metagenomic approach revealed that endolithic microbial diversity differed between coral species but not within compartments of the same coral host. Endolithic communities were more diverse in *G. edwardsi* than in *P. lutea*, with differentially more abundant Deltaproteobacteria in *G. edwardsi* and Cyanobacteria in *P. lutea*

### Endolithic microbiomes of *G. edwardsi* are functionally more diverse and redundant

**Fig. 4:**
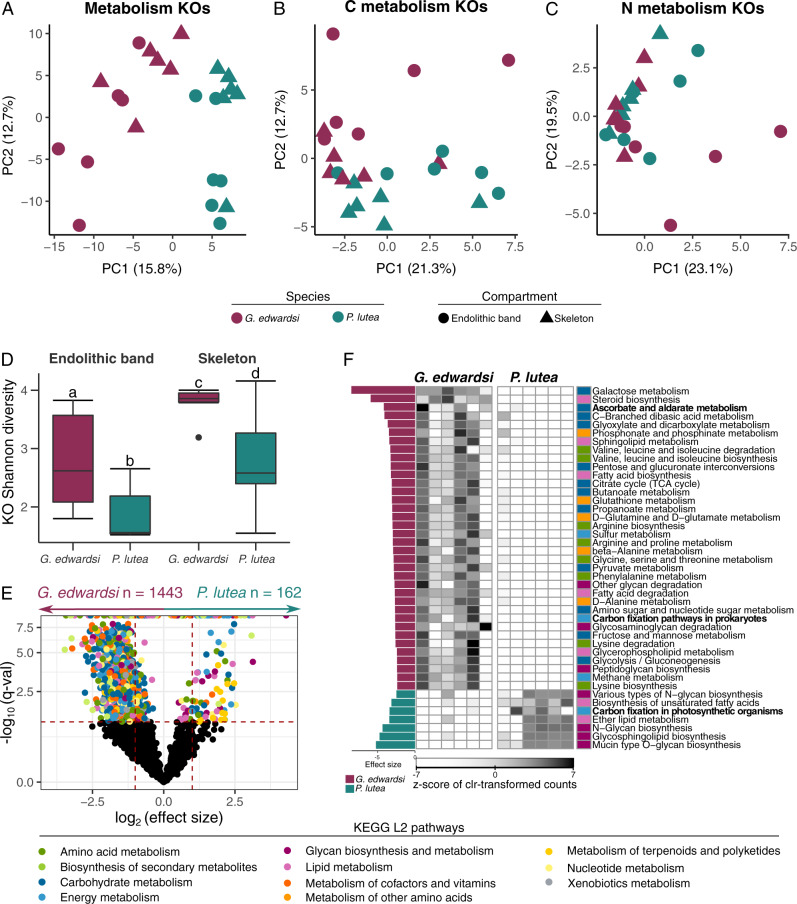
Metagenomic functional diversity in skeletal compartments of *G. edwardsi* and *P. lutea*. Ordination plots based on the composition of KEGG orthologs (KOs) involved in **A** overall metabolism, **B** carbon metabolism, and **C** nitrogen metabolism. **D** Shannon diversity of metabolism KOs. Significant differences between species and skeletal compartments are denoted by letters above the boxes. **E** Volcano plots depicting differentially abundant KEGG orthologs (KO) involved in metabolism between *G. edwardsi* and *P. lutea*. Each dot represents a KO and differentially abundant KOs are colored by metabolic processes. KOs that are not differentially abundant between species are colored in black. Dashed red lines represent thresholds for FDR-adjusted *p* value <  0.05 and effect sizes (1 and −1) represented by the Beta statistic obtained from ANCOM-BC. **F** Differentially abundant carbon metabolism KEGG L3 pathways between *G. edwardsi* and *P. lutea*. Clr-transformed pathway counts were plotted and z-normalized by KEGG L3 pathways (rows). Left bars indicate the effect size represented by the W ANCOM-BC statistic and colors represent the coral species in which the process was enriched. Colored boxes represent the affiliation to KEGG L2 pathways.

We assessed the metabolic diversity of endolithic microbiomes using a gene-centric approach based on the composition of KEGG Orthologs (KOs) involved in metabolism (KEGG map 01100) between coral species and skeletal compartments. Metabolic diversity was significantly different between species in the endolithic band and the skeleton (PERMANOVA, *p*_adj_ < 0.05), but not significantly different between compartments of the same species (Fig. 4A and Table S9). Species clustering of KOs was evident in ordination plots of overall metabolism (Fig. 4A) and C metabolism (Fig. 4B), while no clustering by species was observed in ordination plots based on N metabolism (Fig. 4C). *G. edwardsi* was metabolically more diverse than *P. lutea* in both compartments, and the skeleton was more diverse than the endolithic band in both species (Fig. 4D and Table S10).

A higher number of KOs and L3 KEGG pathways were enriched in *G. edwardsi* in comparison to *P. lutea* (Fig. 4E and Tables S7 and S10). L3 KEGG pathways enriched in *G. edwardsi* in comparison to *P. lutea*, were mainly associated with carbohydrate (21.1%), amino acid (14.0%), and cofactors and vitamin metabolism (12.3%) (Fig. 4F). Contrastingly, *P. lutea* was enriched in processes involved in the biosynthesis of other secondary metabolites (30.8%), glycan biosynthesis and metabolism (30.8%), and lipid metabolism (15.4%) compared to *G. edwardsi* (Fig. 4F). We performed a *Selbal* analysis to identify functional signatures predictive of coral species for each compartment. “Carbon fixation in photosynthetic organisms” was the process with the largest discriminatory effect between coral species and was predictive of the *P. lutea* endolithic band and skeleton microbiomes, while “Ascorbate and aldarate metabolism” was predictive of *G. edwardsi* endolithic band microbiomes and “Galactose metabolism” was predictive of *G. edwardsi* skeleton microbiomes (Figure S12). Of note, the “Carbon fixation in photosynthetic organisms” pathway includes reactions that fix CO_2_ to organic compounds by the reductive pentose phosphate cycle (i.e., Calvin cycle), unlike the “Carbon fixation pathways in prokaryotes” that includes pathways used by autotrophic bacteria and archaea.

**Fig. 5:**
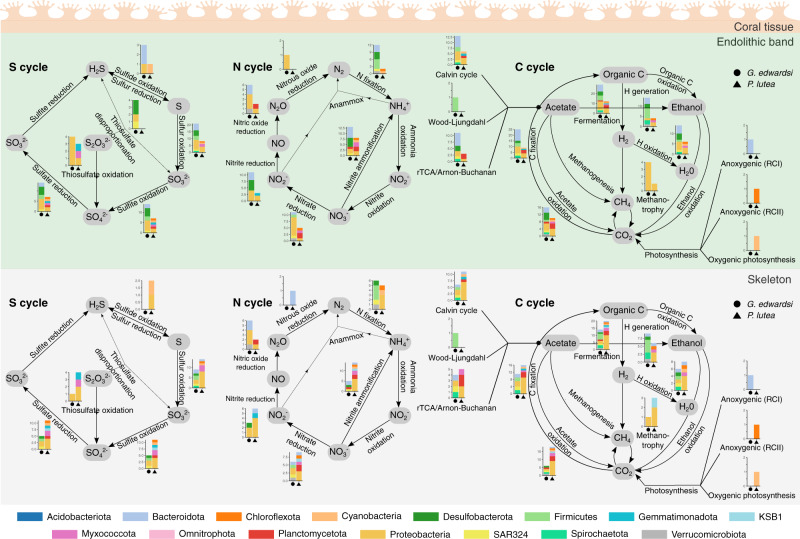
Taxonomic distribution of central N, S, and C pathways in endolithic metagenome-assembled genomes (MAGs). For each MAG, only pathways with more than 70% completeness were considered. Barplots show the number of MAGs from each phylum that were assigned to each reaction in *G. edwardsi* (circles) and *P. lutea* (triangles).

We investigated the prevalence of different prokaryotic carbon fixation pathways as well as their relative contribution in each coral species and compartment (Figure S13). Carbon fixation pathways in *G. edwardsi* were dominated by the reductive acetyl-CoA pathway (Wood-Ljungdahl pathway), followed by the reductive citric acid cycle (Arnon-Buchanan cycle), the Calvin-Benson cycle, and the 3-hydroxypropionate bi-cycle (Figures S13C-F). In *P. lutea* the reductive pentose phosphate cycle (i.e., Calvin-Benson cycle) of eukaryotic and cyanobacterial origin was prevalent, representing more than 50% of the relative contribution to total carbon fixation genes in the endolithic band (Figure S13B, S13F). Bacterial MAGs revealed that prokaryotic carbon fixation pathways had a broader phylogenetical distribution in *G. edwardsi* compared to *P. lutea*. In *G. edwardsi*, members from 6 phyla (Bacteroidota, Desulfobacterota, Firmicutes, Proteobacteria, SAR324, and Spirochaetota) harbored at least 70% of the genes involved in carbon fixation in prokaryotes, while in *P. lutea* only 1 phylum (Proteobacteria) harbored the genes involved in the Arnon-Buchanan cycle (Fig. 5 and S14).

MAGs enriched in *G. edwardsi* revealed a higher functional redundancy than in *P. lutea*. Indeed, 92% of the processes found in the endolithic band were present in more MAGs in *G. edwardsi* than in *P. lutea* and each of these processes was represented on average by 2.6 times more taxonomic groups in *G. edwardsi* compared to *P. lutea*. Conversely, 83% of the processes found in the skeleton were present in more MAGs in *P. lutea* than in *G. edwardsi* and each of these processes was represented on average by 1.8 times more taxonomic groups in *P. lutea* compared to *G. edwardsi*. (Fig. 5). Processes including photosynthesis, nitrogen fixation, and sulfate reduction were enriched in the binned fraction of *P. lutea* in comparison to *G. edwardsi*, whereas methanotrophy, thiosulfate metabolism, sulfur oxidation, and nitrate reduction were enriched in *G. edwardsi*’s binned fraction in comparison to *P. lutea*. Anoxygenic photosynthesis was enriched in the skeleton of both species and nitrite ammonification was enriched in the endolithic band of both species (Fig. 5, S14, and S15).

In summary, both gene-centric and genome-centric metagenomic results revealed that *G. edwardsi* harbors endolithic microbiomes with greater and more redundant functional diversity than *P. lutea*. These findings also indicate that genes involved in carbon metabolism, in particular carbon fixation, differed between coral hosts, with anaerobic prokaryotic carbon fixation being more abundant in *G. edwardsi* and aerobic carbon fixation (i.e., Calvin-Benson cycle) being more abundant in *P. lutea*.

### Taxonomic and functional diversity of skeletal compartments under heat stress

There was no significant difference in the overall taxonomic composition or alpha diversity between control and heat stress samples in either *G. edwardsi* or *P. lutea* (Figure S16A, B). Despite the lack of significant differences in the overall microbiome composition between treatments, we identified differentially abundant families between treatments (Table S7). Microbial communities associated with the skeleton of *G. edwardsi* under heat stress changed the most compared to controls, with 268 differentially abundant prokaryotic and eukaryotic families identified, indicating a higher microbial response to heat in that compartment (Figure S17). These differentially abundant families included members of marine basidiomycete and ascomycete Fungi, specifically the classes Agaricomycetes (*n* = 37) and Sordariomycetes (*n* = 20), respectively, which significantly decreased with heat stress, while bacterial families affiliated to the Desulfobacterota phyla (*n* = 7), including Desulfomicrobiaceae and Desulfuromonadaceae, significantly increased (Table S7). In line with this result, ASVs belonging to the phylum Desulfobacterota and Gammaproteobacteria class were also enriched in the 16S rRNA gene amplicon dataset during heat stress (Table S7).

Similarly, we did not detect significant differences in the overall microbial functional composition or alpha diversity of KEGG orthologs between control and heat stress endolithic microbiomes (Figure S16C, D and Tables S9,10), consistently with fewer differentially abundant KOs and pathways between treatments (Figure S18 and Table S7). *P. lutea* was enriched with KOs involved in glycolysis/gluconeogenesis and oxidative phosphorylation in comparison to *G. edwardsi*. In contrast, *G. edwardsi* had a more diverse metabolic response to heat stress, which included KOs involved in methanogenesis (i.e., formation of methane from CO_2_), prokaryotic carbon fixation (i.e., 3-Hydroxypropionate bi-cycle and the reductive citrate cycle, acetyl-CoA), antenna proteins (i.e., phycocyanin synthesis and phycobilisome light-harvesting complex), and sulfur metabolism (i.e., thiosulfate metabolism and dissimilatory sulfate reduction) (Figure S18).

### *P. lutea* assimilates more carbon and nitrogen

Given the differences in metabolic potential identified between both coral species, we employed nanoscale secondary ion mass spectrometry (NanoSIMS) to determine whether carbon and nitrogen assimilation differed in the skeletal compartments of both coral species. Entire coral cross sections were initially analyzed, but the carbonated skeleton had a negative impact on the NanoSIMS analysis, resulting in a very low signal for all the isotopic species of interest (^12^C, ^13^C, ^14^N, and ^15^N). Although low conductivity of biomineralized carbonates has previously been reported [85], the patterns observed here were very pronounced (Figures S1H–O), and the data acquired using this approach were not usable. Specifically, we observed a complete lack of NanoSIMS signal from skeletal surfaces and greatly attenuated signals (together with spatial artifacts, as shown in Figure S1H) in neighboring skeletal pores infiltrated with resin. We, therefore, used an alternative approach to prepare the samples for NanoSIMS, decalcifying separately the tissue and endolithic compartment sections followed by resin embedding of each compartment.

**Fig. 6:**
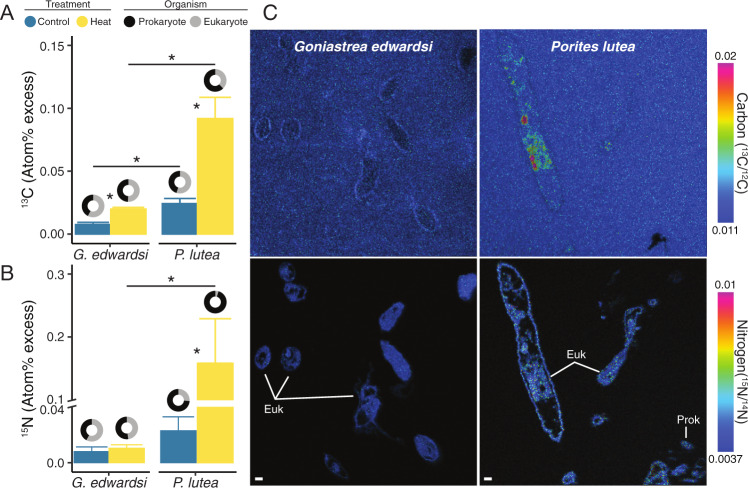
Carbon and Nitrogen uptake by endolithic microorganisms under heat stress. **A** ^13^C and **B** ^15^N assimilation in *G. edwardsi* and *P. lutea*. Donut charts represent the proportion of ^13^C and ^15^N assimilated by prokaryotes or eukaryotes. **C** Representative NanoSIMS images showing ^13^C and ^15^N assimilation in prokaryotic and eukaryotic cells. Scale bar: 1 µm.

Results derived from this second approach revealed that after incubation with ^13^C-bicarbonate and ^15^N-ammonium, endolithic microorganisms in *P. lutea* were on average 3.2- and 2.9-times more enriched in ^13^C and ^15^N, respectively, than those in *G. edwardsi* (Fig. 6A, B, Table S11). Endolithic microorganisms were characterized as eukaryotes if cells exceeded 3 μm and as prokaryotes if cells were smaller than that threshold. Filamentous endolithic eukaryotes in *P. lutea*, potentially belonging to Ostreobiaceae (Ulvophyceae) or Aspergillaceae (Eurotiomycetes; Fig. 6C), were on average 2.8-times more enriched in ^13^C compared to those in *G. edwardsi* (Kruskal-Wallis test, *p* < 0.01; Figure S19). Similarly, endolithic prokaryotes in *P. lutea* were on average 3.1-times more enriched in ^13^C than those in *G. edwardsi* (Kruskal–Wallis test, *p* < 0.01; Figure S19). Conversely, ^15^N assimilation was barely detectable and not statistically different between coral species when eukaryotes and prokaryotes were considered separately. In addition, coral tissue in *P. lutea* exhibited a significantly larger enrichment in ^13^C but a significantly lower enrichment in ^15^N compared to *G. edwardsi* (Kruskal–Wallis test, *p* < 0.01; Figure S19), although the enrichment of their Symbiodiniaceae was not statistically different.

Although only subtle differences were identified in microbial functional diversity between control and heat stress treatments using our metagenomic approach, the assimilation of ^13^C-bicarbonate by microorganisms from the endolithic band was significantly higher under heat stress in both coral species compared to control conditions (Kruskal-Wallis test, *p* < 0.01). Indeed, a 2.6- and a 3.8-fold increase in carbon assimilation were recorded in endoliths from *G. edwardsi* and *P. lutea*, respectively (Fig. 6A). During heat stress, the endoliths in *P. lutea* assimilated 4.6-fold more ^13^C than in *G. edwardsi* (Fig. 6A). Although heat stress had no effect on ^15^N assimilation in *G. edwardsi* (Fig. 6B), endoliths in heat-stressed *P. lutea* assimilated 82 times more ^15^N than in control colonies and 184 times more than *G. edwardsi* at 33 °C (Fig. 6B). More specifically, eukaryotic endoliths in *P. lutea* assimilated 12.4 times more ^15^N, while prokaryotic cells assimilated 88.2-times more under heat stress than in the control fragments (Kruskal-Wallis test, *p* < 0.01; Figure S19). This large increase in ^15^N assimilation in *P. lutea* endoliths under stress is in stark contrast with the relatively low and similar levels of assimilation in the endoliths harbored by the control colonies of both species. Meanwhile, ^13^C and ^15^N assimilation both significantly decreased under heat stress in the coral tissue from *P. lutea* (Kruskal–Wallis test, *p* < 0.01; Figure S19C).

To sum up, endolithic microorganisms in *P. lutea* assimilated more carbon and nitrogen than those from *G. edwardsi* under ambient conditions, and these differences were exacerbated further by heat stress. NanoSIMS data therefore indicated that differences in metabolic potential between the endolithic communities of the two coral species contributed to drastic dissimilarities of nutrient uptake, highlighting the putative contribution of endolithic microbiomes to heat stress susceptibility, as further discussed below.

## Discussion

Coral bleaching is the primary driver of global reef loss, prompting an urgent need to advance understanding of the mechanisms underlying bleaching susceptibility to mitigate the effects of ocean warming [5, 38]. We are only beginning to understand the role of the endolithic microbiome in coral holobiont functioning and its putative role in mitigating the effects of bleaching, despite the coral skeleton being the largest microbial compartment by means of volume [18]. Previous studies have shown that endolithic microbes can supply the coral tissues with nutrients [29, 30], suggesting that these endolithic communities may act as a “rescue compartment” to supply fixed carbon to compensate for the loss of Symbiodiniaceae during bleaching. Still, we remain naïve with regard to a definitive link between endolithic microbiome structure, functional potential, and coral bleaching susceptibility. To overcome methodological limitations that hamper the exploration of skeletal compartments, we integrated emerging technologies, i.e., single-cell imaging of nutrient assimilation with metagenomic interrogation to characterize endolithic microbial functioning and their potential contribution to bleaching susceptibility.

### Metabolic strategies in endolithic microbiomes of two coral species with different bleaching susceptibilities

Endolithic microbiomes were distinct between coral species (i.e., *P. lutea* and *G. edwardsi*), but did not differ across endolithic compartments within the same species (Figs. 3F and 4A, Table S5 and S9). The lack of microbial differences between compartments was attributed to high variability across replicates. Therefore, we encourage future studies to include a greater number of replicates to tease apart microbiome differences between the different compartments. In contrast to previous approaches based on ribosomal gene amplicon sequencing [20, 86], our comparative metagenomic approach allowed for a direct comparison across phylogenetically distant groups of endoliths and revealed that bacterial communities accounted for the majority (~75%) of the DNA content in metagenomic samples derived from coral skeleton (Figure S4). Coral host genomic material typically accounts for the majority of the sequenced DNA in tissues [87, 88], but we believe the low host coverage in our study is due to the low coral biomass compared to microbial cells in the skeleton, together with our targeted sampling of the skeletal compartments. One of the most striking observations was the low abundance (<1% of the metagenomic community) of the common microalgal family Ostreobiaceae, often-considered a prominent member of the endolithic band (Fig. 3C). This is consistent with previous reports of low amplicon read numbers affiliated to this taxon [20, 32]. Our findings suggest that differences in endolithic microbiomes between the two coral species were largely explained by the bacterial composition, with *G. edwardsi*’s endolithic microbiomes being enriched with families belonging to the strictly anaerobic Desulfobacterota, including Desulfobacteraceae and Desulfovibrionaceae, and *P. lutea*’s endolithic microbiomes enriched with aerobic cyanobacterial families including Cyanobacteriaceae and Phormidesmiaceae (Fig. 3B and S5B). The prevalence of strictly anaerobic bacteria co-occurring with diverse microalgal assemblages in *G. edwardsi*’s endolithic microbiomes has previously been reported in skeletal compartments [34, 89–91] and supports the existence of micro-niches that protect strict anaerobes from strong daily oxygen oscillations ranging from 10% to 210% air saturation [92].

Functional profiles, particularly those related to carbon metabolism, were largely driven by the relative proportion of anaerobic vs aerobic bacterial taxa. In this regard, the functional signatures that best discriminated endolithic microbiomes were characterized by genes involved in ascorbate metabolism in *G. edwardsi* and genes involved in the Calvin-Benson cycle in *P. lutea* (Figure S12). Ascorbate metabolism includes the ROS-scavenging enzyme ascorbate peroxidase, involved in protection against O_2_ toxicity and reported to be an important mechanism for alleviating photodamage in eukaryotic microalgae, including Symbiodiniaceae, and higher plants during high light intensities [93–95]. These findings, while preliminary, raise the possibility that endolithic microbiomes may be more effective at alleviating heat-induced oxidative stress in *G. edwardsi* than in *P. lutea*. On the other hand, *P. lutea* skeletal compartments were enriched with genes associated with the Calvin-Benson cycle, whereas *G. edwardsi*’s skeletal compartments were enriched in three carbon fixation pathways exclusive to prokaryotes (Figure S13F). While the Calvin-Benson cycle is the most important contributor to autotrophic CO_2_ fixation in the ocean, prokaryotic CO_2_ fixation has also shown to be of high significance, especially in symbiosis in extreme environments [96]. In contrast to the Calvin-Benson cycle, most prokaryotic carbon fixation reactions found in the skeletal compartments of *G. edwardsi* are oxygen-sensitive and can only be carried out in microaerophilic (i.e., Arnon-Buchanan cycle) or strict anaerobic conditions (i.e., Wood–Ljungdahl pathway). These pathways require less ATP to sustain growth [97, 98], but contain energetically challenging reactions that require a low redox potential and high inorganic carbon concentrations [99]. As a result of such energetic constraints, primary production could be lower in anaerobic chemolithotrophy compared to the Calvin-Benson cycle. This aligns with the higher ^13^C assimilation measured in the skeleton of *P. lutea*, which indicates greater levels of primary production.

It is now widely accepted that higher functional diversity and redundancy (i.e., the capacity of one species to functionally compensate for the loss of another) [100, 101] are critical to ecosystem functioning and resilience [102, 103]. We found that endolithic microbiomes in *G. edwardsi*’s were overall more diverse (taxonomically and functionally) as well as functionally more redundant in comparison to endolithic microbiomes in *P. lutea* (Figs. 3E and 4D). This suggests that *G. edwardsi*’s endolithic microbiomes may have a greater repertoire of mechanisms to respond to environmental disturbances, while being less likely to disrupt holobiont homeostasis as a consequence of the loss of microbial functional groups. In this context, we argue that the inability to maintaining holobiont functionality is presumably a crucial aspect of bleaching susceptibility, as evidenced by the dependence of the coral host on organic carbon translocated from its photosynthetic algal symbionts [104]. Besides bleaching susceptibility, microbiomes with a diverse functional potential have been proposed as an adaptive trait enabling corals to persist in highly polluted waters [105]. For instance, endolithic communities capable of utilizing diverse metabolic substrates (e.g., pollutants or excess nutrients) could contribute to maintaining low nutrient concentrations in the overlaying coral tissue. Thus, functionally diverse endolithic communities may increase holobiont environmental resilience, potentially explaining previous reports that identified corals from the genus *Goniastrea* as environmentally resilient [106, 107]. Our results also argue that higher metabolic diversity (as estimated in *G. edwardsi*) does not necessarily translate into higher metabolic productivity, but that metabolic versatility might be a critical trait affecting holobiont functioning under stress.

### Heat stress stimulates endolithic metabolism

After 17 days of heat stress, *P. lutea* visibly bleached whereas *G. edwardsi* showed no visible signs of stress, such as symbiont loss. Besides the apparent differences in bleaching susceptibility, taxonomic and functional profiles were only marginally affected by heat stress and a relatively low number of differentially abundant families were identified between control and heat stress samples (Figures S16, S17). Interestingly, some of these families contributed considerably to the observed differences between coral species (e.g., members of the Desulfobacterota, Figure S9), indicating that heat stress may have exacerbated differences in endolithic communities between these two coral species and implying the existence of species-specific microbial responses to thermal stress. These findings also highlight the stability of these endolithic communities, consistent with the fairly stable environment in which they live and align with prior evidence supporting the stability of endolithic communities under natural gradients of high pCO_2_ [32]. Our results therefore suggest that endolithic communities respond to heat stress by increasing cellular metabolism, whereas microbial community composition remains unaffected, as shown previously for coral reef microbial communities under environmental change [108, 109].

Interestingly, we showed that ^13^C assimilation was enhanced by heat stress in the endolithic band of both coral species, but only *P. lutea* assimilated significantly more ^15^N during heat stress (Fig. 6A-B). Higher endolithic ^15^N assimilation in heat-stressed *P. lutea* may be due to increased access to seawater nutrients as a result of Symbiodiniaceae expulsion from the coral tissue. This could in turn stimulate the growth of endolithic microbes, which may explain previous reports of rapid endolithic proliferation following bleaching events [29, 110].

### Putative role of the endolithic microbiome in holobiont stress tolerance

**Fig. 7:**
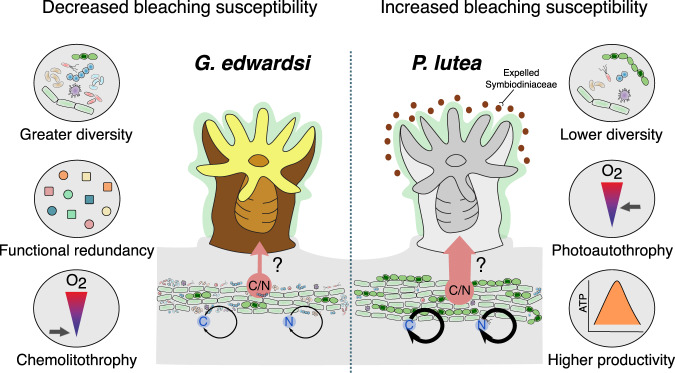
Proposed model on the effects of endolithic microbiome functional traits in holobiont nutrient cycling and bleaching susceptibility. Endolithic microbiomes of the bleaching-resistant *G. edwardsi* were characterized by greater taxonomic diversity in conjunction with greater functional diversity and redundancy, which contrasted with endolithic microbiomes associated with the bleaching-sensitive *P. lutea*. C and N assimilation was greater in *P. lutea* than *G. edwardsi* (represented by black arrows), likely explained by the dominance of aerobic photoautotrophy over chemolithotrophy, respectively. Less C and N are expected to be translocated from the skeleton to the host tissues in *G. edwardsi* than in *P. lutea* (red arrows).

Coral hosts have been proposed to be capable of promoting specific microbial associations to rapidly alleviate environmental stress [111–113], but very little is known about the microbial traits that support host acclimatization to environmental stress, and which trade-offs occur between stress tolerance and holobiont fitness. Our findings show that two coral species with different skeletal physical properties harbor distinct endolithic microbiomes, resulting in contrasting metabolic strategies that may differentially affect bleaching susceptibility (Fig. 7). The bleaching-resistant *G. edwardsi* was associated with a higher taxonomic and functional endolithic diversity dominated by generalist bacteria with higher metabolic flexibility, which resulted in functionally redundant endolithic microbiomes and lower microbial C and N assimilation. In contrast, skeletal compartments of the bleaching-sensitive *P. lutea* harbored a lower taxonomic and functional endolithic diversity dominated by specialist bacteria such as Cyanobacteria and, concomitantly, higher C and N assimilation. We hypothesize that the lower productivity observed in *G. edwardsi*’s endoliths, attributed to the dominance of chemolithotrophs, may result in a constant translocation of C or N of lower magnitude from the skeleton to the host tissues, which may be beneficial in maintaining N limitation, a central feature of the stable coral-algal symbiosis [7, 104]. In contrast, the higher N assimilation observed in *P. lutea*’s endoliths during heat stress could result in increased N translocation and subsequent excess in the tissues. N excess has previously been linked with coral bleaching [114–116], and is known to rapidly disrupt the coral–algal symbiosis during heat stress [104].

We propose a functional link between endolithic microbiome composition, metabolic activity, and bleaching susceptibility, in which the dominance of endolithic photoautotrophs can promote the translocation of excess nutrients to the tissues, which may contribute to Symbiodiniaceae expulsion. Further experimental work in other coral species is required to establish whether the dominance of photoautotrophs is linked to a higher C and N metabolism and whether increased endolithic metabolism is indeed linked to the coral bleaching response. Our findings raise important questions in the context of nutrient cycling and the implications of changes in endolithic metabolism during heat stress. For instance, it would be prudent to elucidate whether highly productive endolithic communities could support the ecological resilience of corals, in particular in the context of overcoming periods of elevated temperature stress. Likewise, it would be important to determine whether less productive endolithic communities favor the re-establishment of the coral-algal symbiosis following bleaching. To better understand whether and how microbes contribute to coral resilience, we must move beyond profiling microbial taxonomic diversity that cannot adequately capture inherent functional potentials. Identifying microbial functional traits that underpin holobiont resilience will be of major importance as they can inform microbiome manipulation approaches to counter the ongoing global loss of coral reefs.

## Supplementary information

Supplementary Figures

Supplementary Tables

## Data Availability

Scripts used are deposited in the GitHub repository https://github.com/ajcardenasb/Coral_endoliths; MAG assemblies are available from Cárdenas and Voolstra [84]. Raw sequencing data are deposited in the NCBI Sequence Read Archive (SRA) under BioProject PRJNA757245 (https://www.ncbi.nlm.nih.gov/bioproject/PRJNA757245).
