# Supplementary material for: Greater functional diversity and redundancy of coral endolithic microbiomes align with lower coral bleaching susceptibility
Source: ISME J. 2022 Jul 15;16(10):2406–20. doi: 10.1038/s41396-022-01283-y (PMC9478130; doi:10.1038/s41396-022-01283-y)
Supplement: Supplementary file 1 — Supplementary Figures [file 41396_2022_1283_MOESM1_ESM.pdf]

**Supplementary figures for:**

# Greater functional diversity and redundancy of coral endolithic microbiomes align with lower coral bleaching susceptibility

Anny Cárdenas[1,2*‡], Jean-Baptiste Raina[3*‡], Claudia Pogoreutz[1,2], Nils Rädecker[1,2,4], Jeremy Bougoure[5], Paul Guagliardo[5], Mathieu Pernice[3], Christian R. Voolstra[1,2‡]

[1]Department of Biology, University of Konstanz, Konstanz 78457, Germany

[2]Red Sea Research Center, Division of Biological and Environmental Science and Engineering, King Abdullah University of Science and Technology, Thuwal 23955, Saudi Arabia

[3]Climate Change Cluster, University of Technology Sydney, Sydney, NSW 2007, Australia

[4]Laboratory for Biological Geochemistry, School of Architecture, Civil and Environmental Engineering, École Polytechnique Fédérale de Lausanne, Lausanne 1015, Switzerland

[5]Centre for Microscopy, Characterisation and Analysis, The University of Western Australia, Perth, WA 6009, Australia

*These authors contributed equally to this work

‡ Corresponding authors:

Anny Cárdenas: anny.cardenas@uni-konstanz.de

Jean-Baptiste Raina: jean-baptiste.raina@uts.edu.au

Christian R. Voolstra: christian.voolstra@uni-konstanz.de

Keywords

Coral skeleton, biogeochemical cycling, symbiosis, autotrophy, metagenomes, NanoSIMS

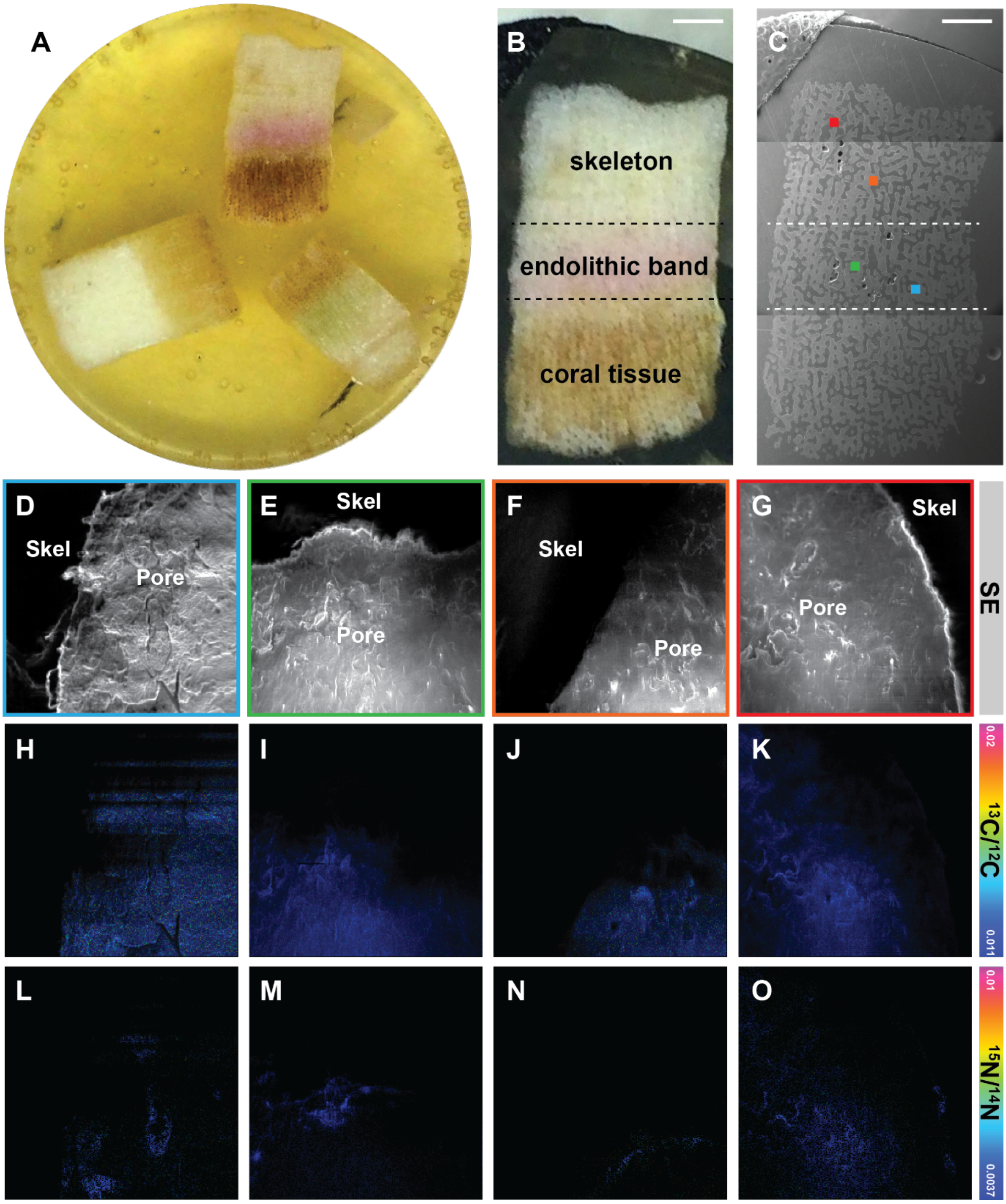


**Figure S1. Initial NanoSIMS procedure, embedding full coral cross sections in resin.** **(A)** Representative resin block with three biological replicates of a given treatment. **(B)** Light microscopy image of a given cross section, with a clear delineation of the coral tissue (brown), endolithic band (red or green) and deeper skeleton (white). **(C)** Electron microscopy image showing the location of the targeted areas for NanoSIMS (colored squares). **(D-G)** Secondary electron image of the targeted areas. Spatial distribution of $^{13}C/^{12}C$ ratio **(H-K)** and $^{15}N/^{14}N$ ratio **(L-O)** using NanoSIMS images. Note the complete lack of signal from the skeleton (black), the striping artifacts in its vicinity, and the overall low signal from the pore area (dark-blue to black, when the resin filling the pore should give a uniform carbon signal).

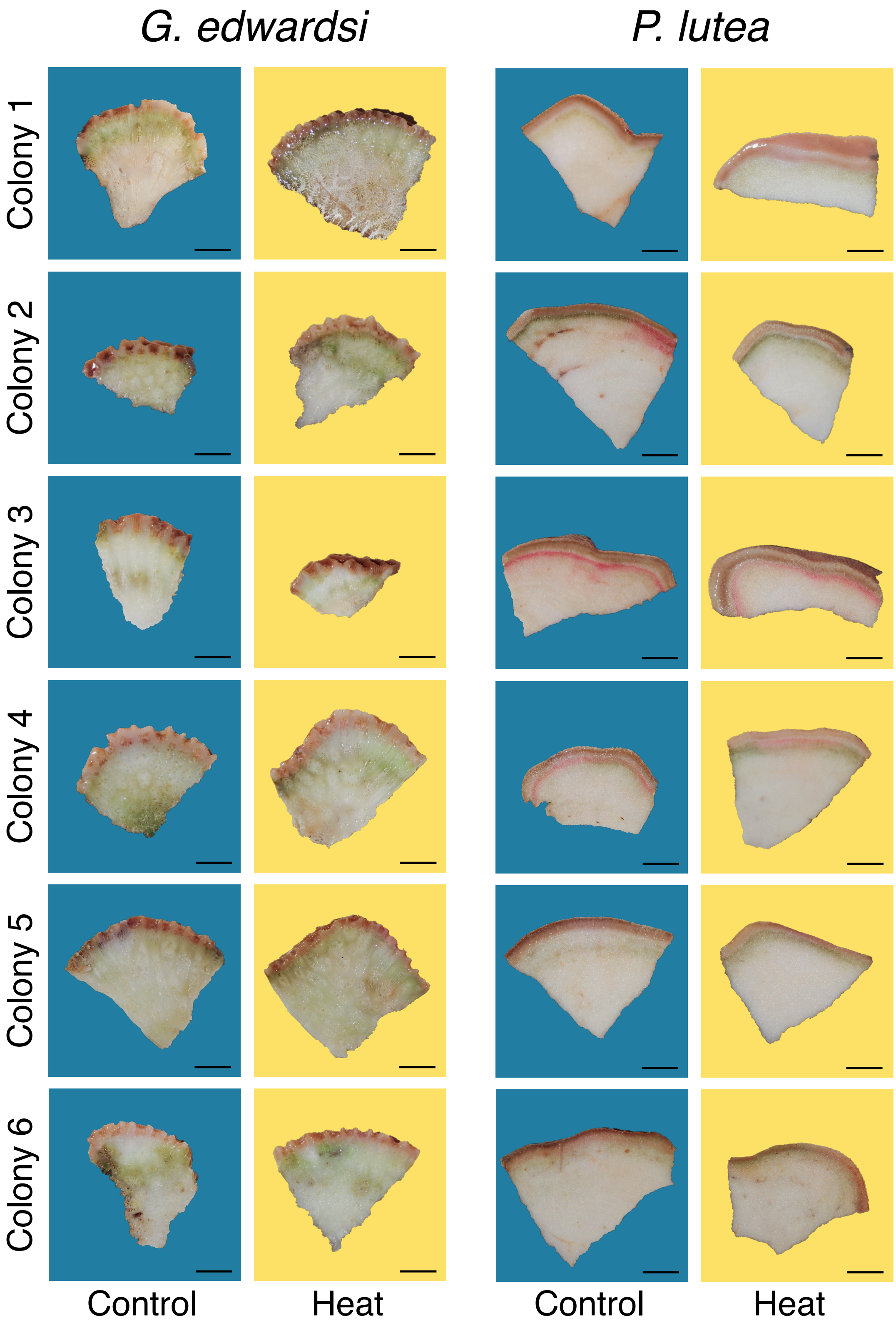


**Figure S2. Overview of skeletal sections of coral colonies.** Photographs correspond to *G. edwardsi* (left) and *P. lutea* (right) at control (blue) and heat stress (yellow) conditions on day 17 of the experiment. All photographs taken under identical light conditions, exposure, and white balance. Scale bar: 1 cm.

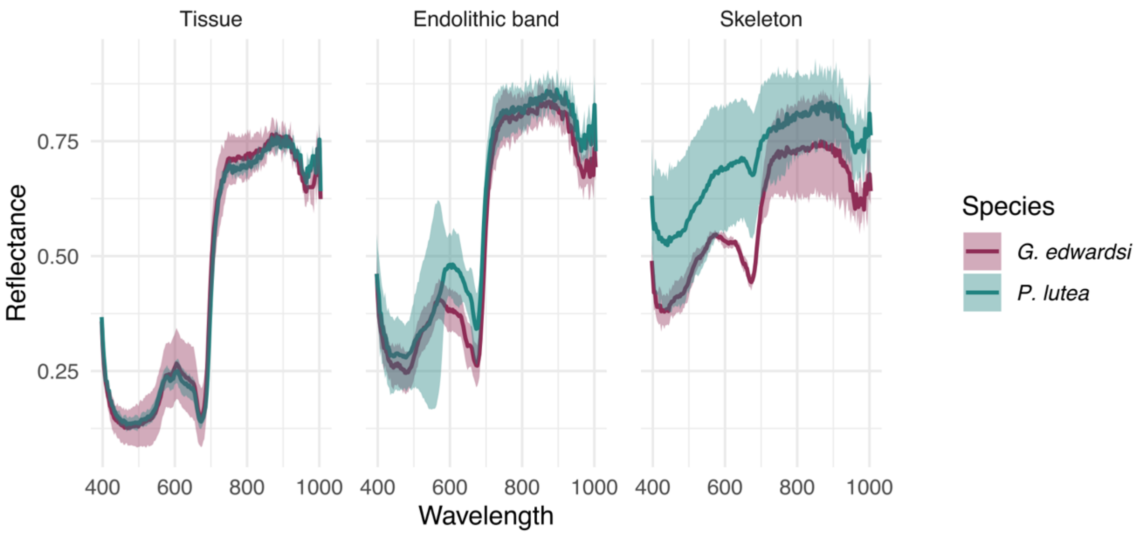


**Figure S3. Hyperspectral profiles between species and skeletal compartments**.

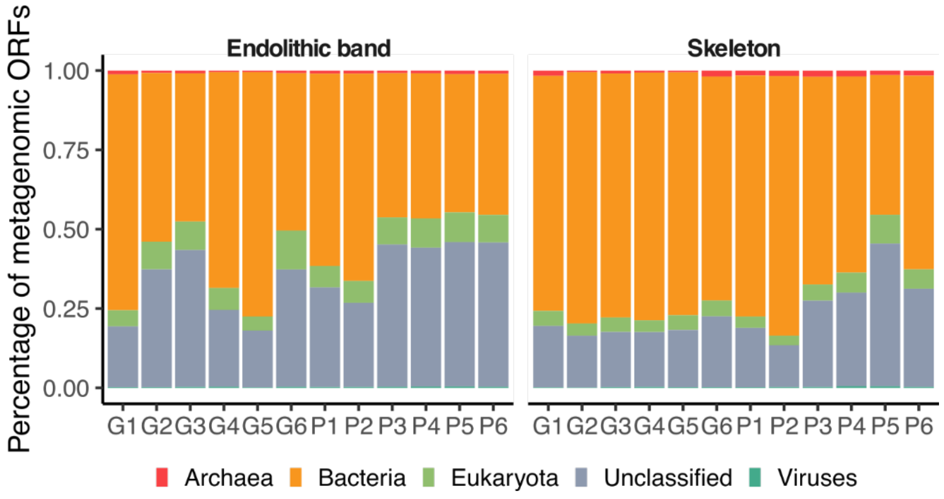


**Figure S4. Overall taxonomic metagenome composition.** Relative abundances of contigs annotated to each superkingdom are represented. *G. edwardsi* samples correspond to G1-G6 and *P. lutea* samples correspond to P1-P6

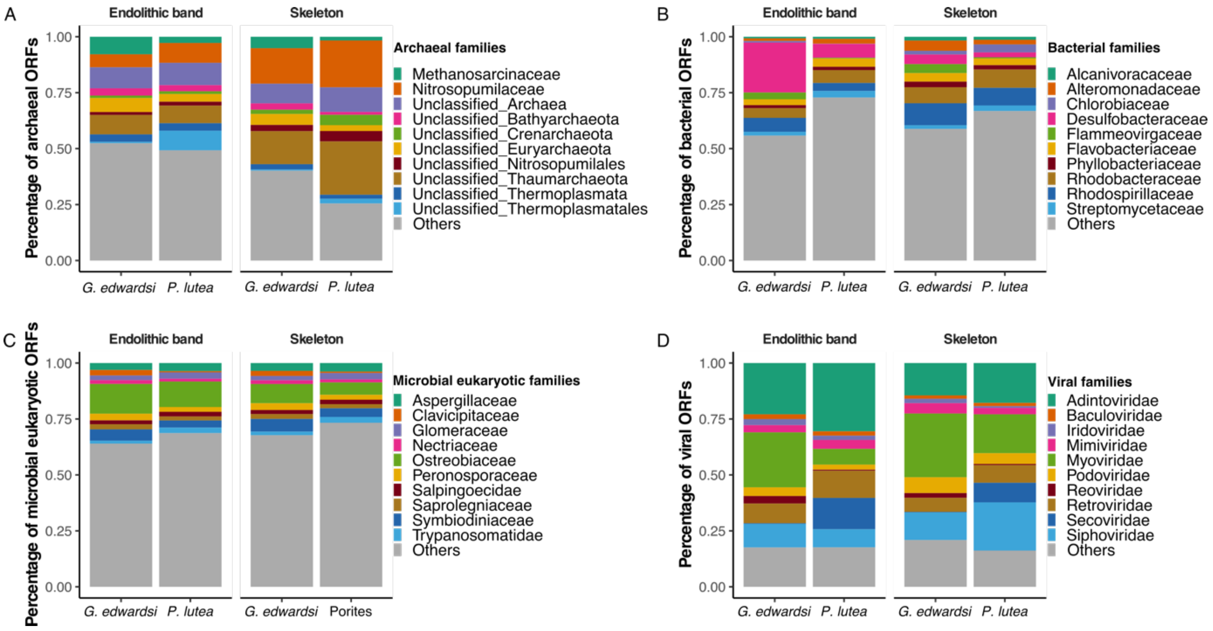


**Figure S5. Top 10 most abundant families per taxonomic domain.** Relative abundances of contigs annotated to the 10 most abundant families are shown. When annotation did not reach the family level, the highest taxonomical rank annotated was used. Less abundant families were aggregated to the "Others" category.

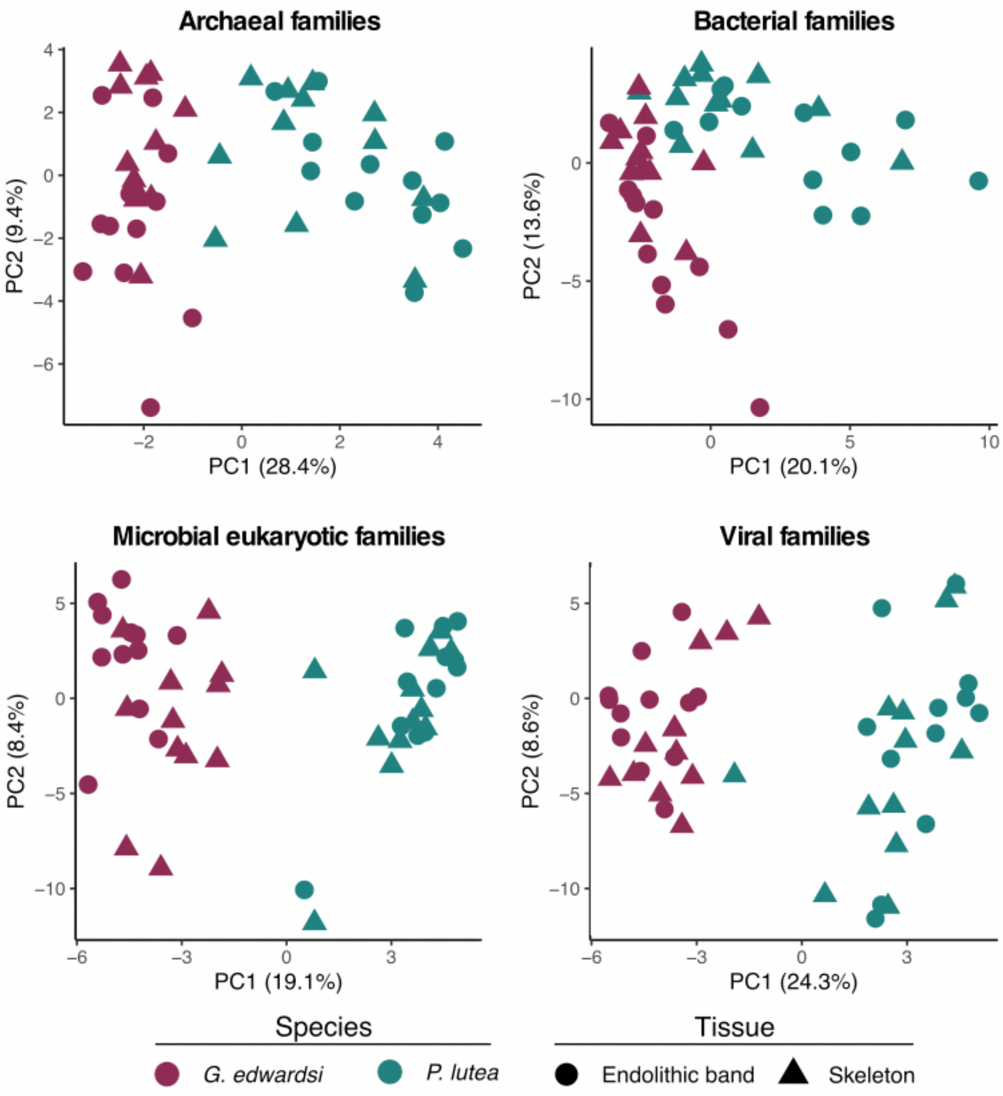


**Figure S6. Principal component analysis of taxonomical families between *G. edwardsi* and *P. lutea*.** Centered-log-ratio-transformed counts were used to calculated Euclidean distances, represented in the 2 first PCs.

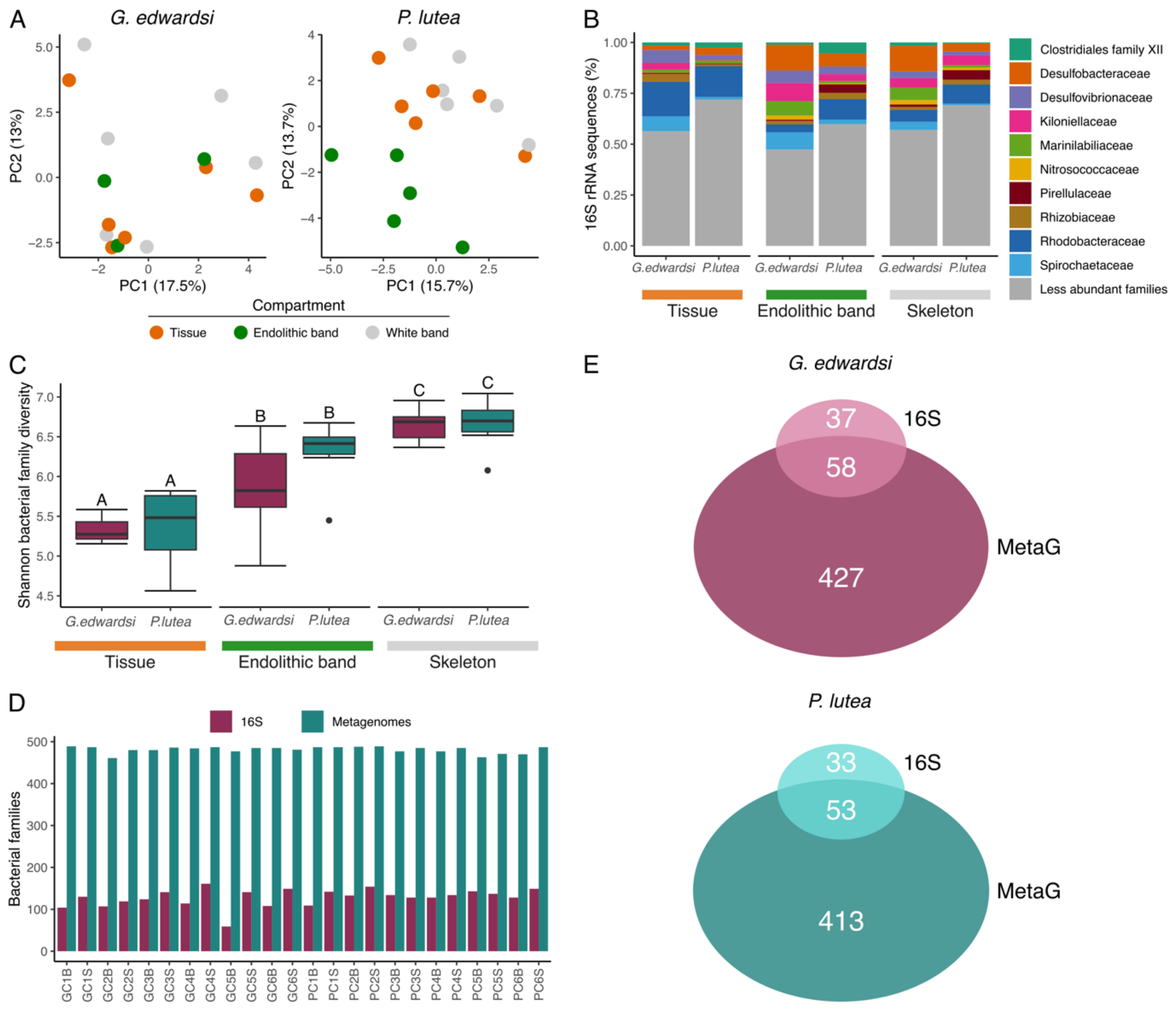


**Figure S7. Bacterial diversity assessed by 16S amplicon sequence variants (ASV). (A)** Principal component analysis of ASV clr-transformed counts based on Euclidean distances. **(B)** 10 most abundant bacterial families across species and tissues. Relative abundances of ASVs annotated to the 10 most abundant families are shown. Less abundant families were aggregated to the "Others" category. **(C)** Shannon bacterial family diversity calculated from rarefied counts. Significant differences between species are denoted by letters above the boxes. **(D)** Observed number of bacterial families on each sample calculated from rarefied counts comparing 16S rRNA and metagenomic libraries. **(E)** Venn diagrams of observed bacterial families found in 16S and metagenomic libraries. Families with counts < 50 were not considered.

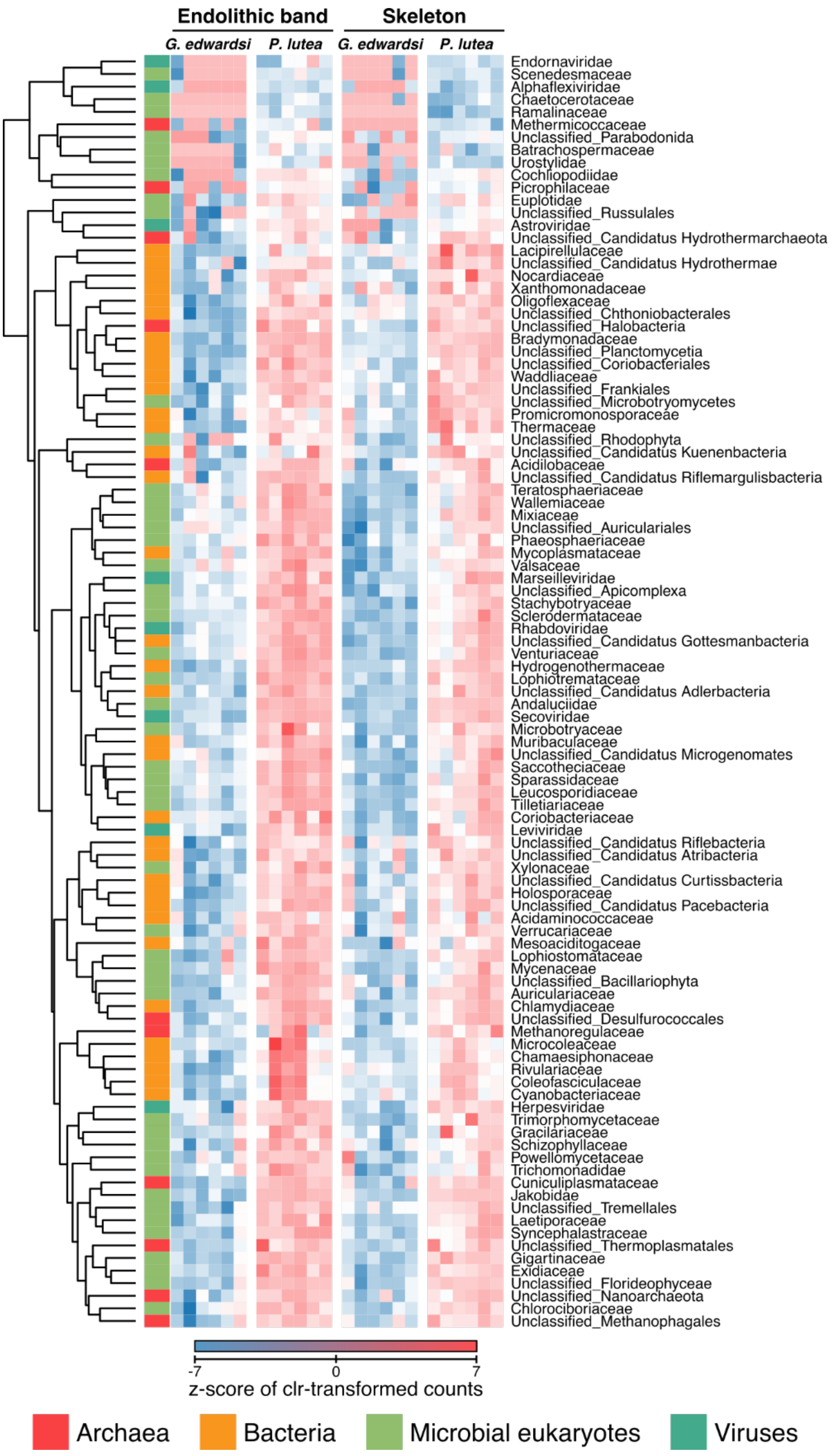


**Figure S8. Differentially abundant metagenomic families between *G. edwardsi* and *P. lutea*.** clr-transformed counts were plotted and z-normalized by taxa (rows). Only families with effect sizes (ANCOM Beta statistic) > 5 were included in the heat map.

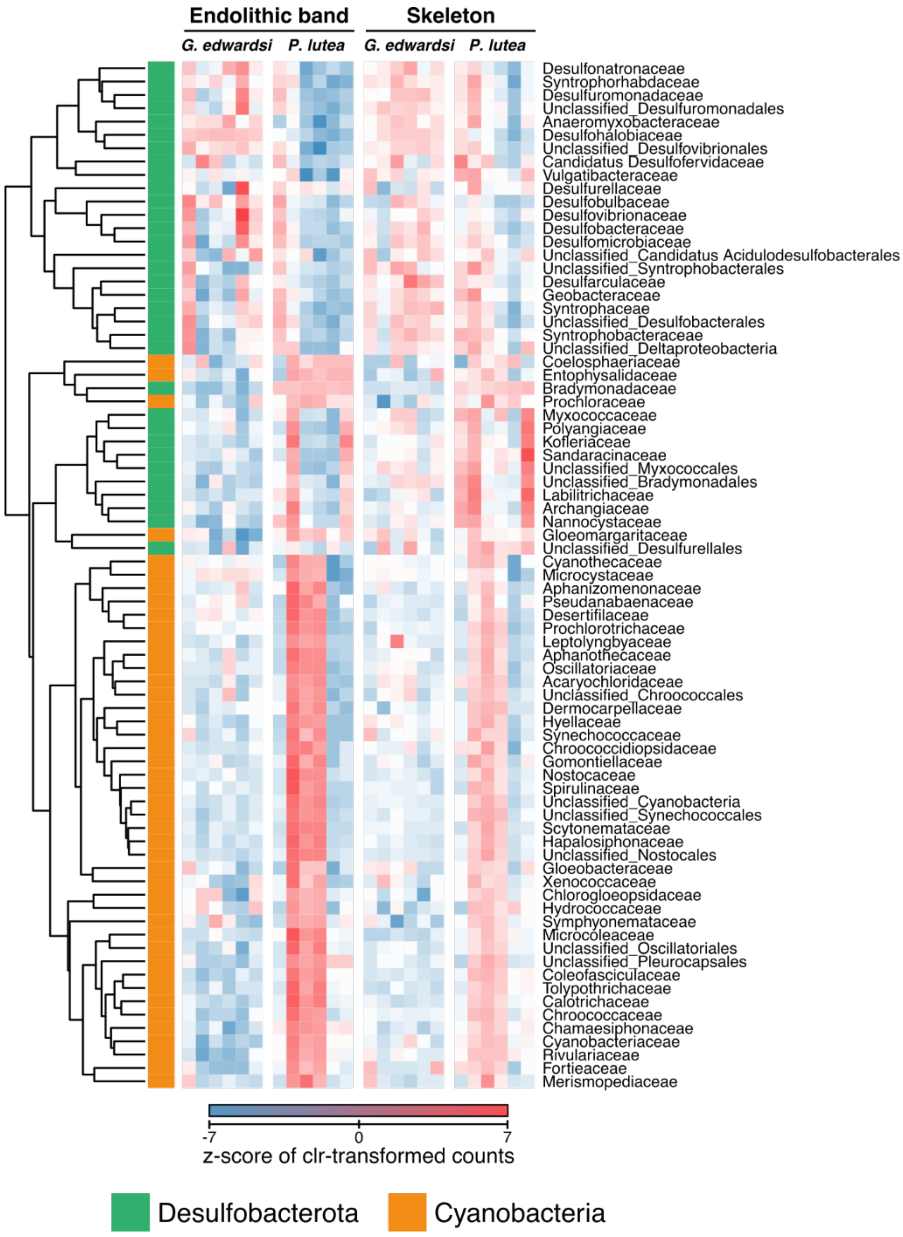


**Figure S9. Differentially abundant metagenomic families of the phyla Desulfobacterota and Cyanobacteria between *G. edwardsi* and *P. lutea*.** clr-transformed counts were plotted and z-normalized by taxa (rows).

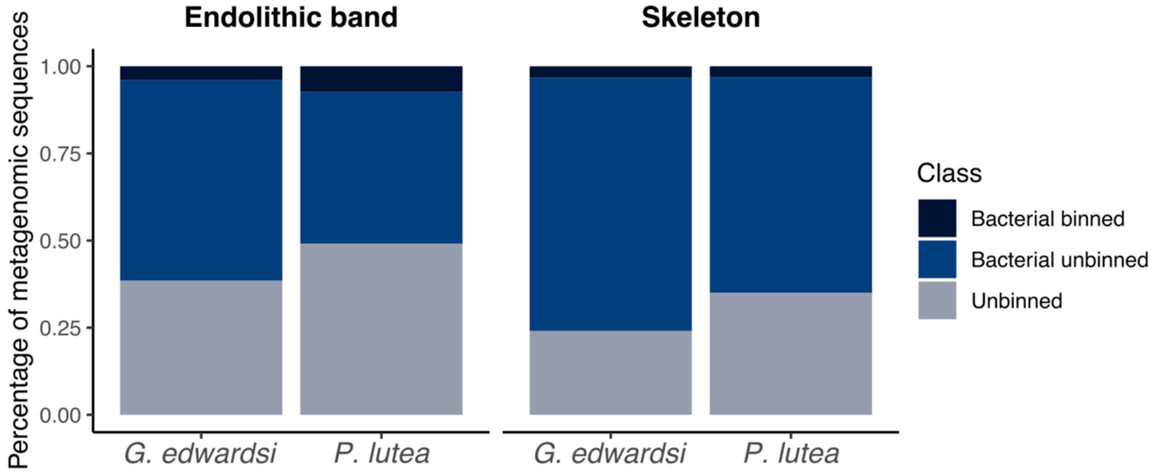


**Figure S10. Contig recruitment during metagenomic binning.** MAG abundances were calculated from the total reads that mapped to the assembly and bacterial reads were estimated from Kaiju's output.

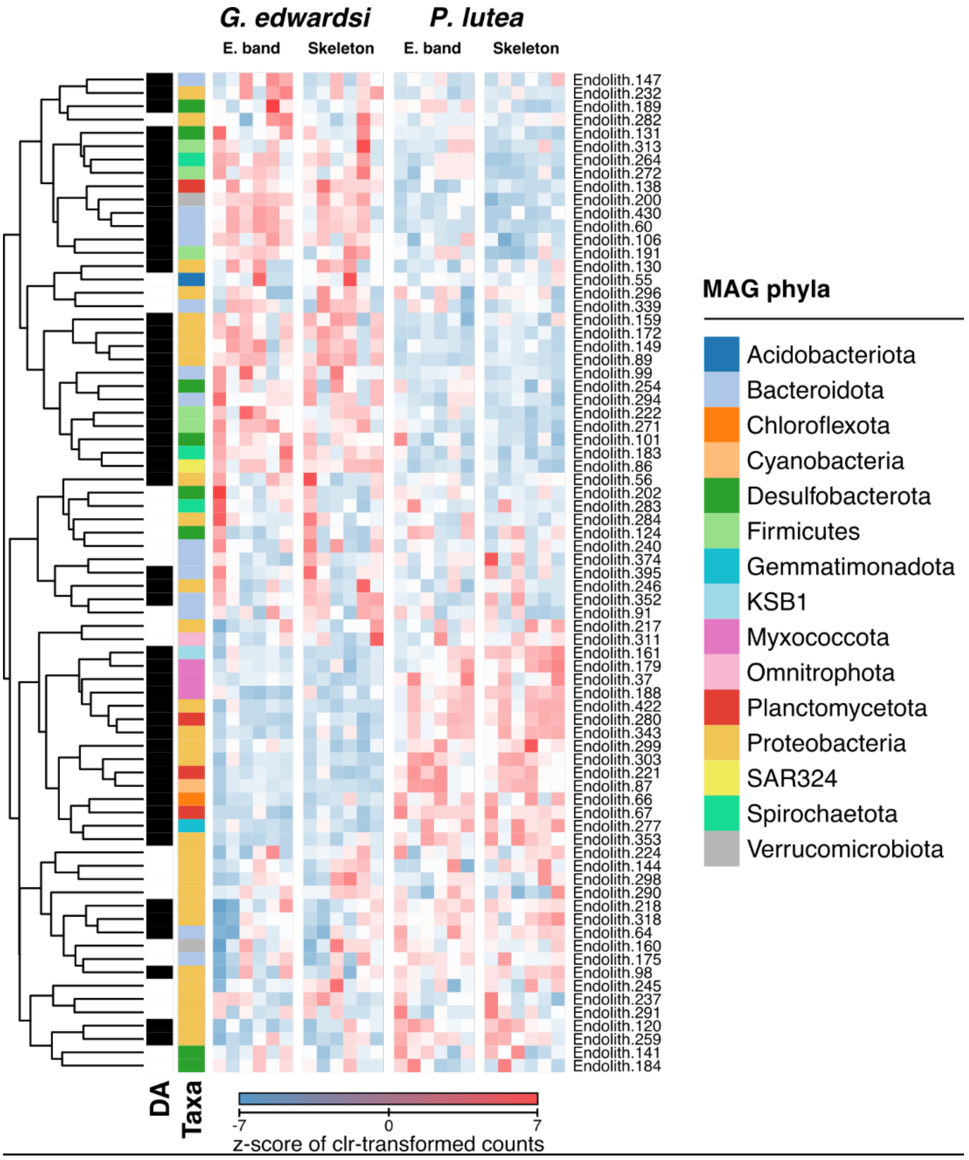


**Figure S11. Metagenome-assembled genomes (MAG) binned from *G. edwardsi* and *P. lutea*. (C)** MAGs abundance across samples and tissues. Abundances correspond to the clr-transformed counts that were z-normalized by MAGs (rows). Taxonomic affiliation of each MAG at the Phylum level is represented in colored squares and differentially abundant (DA) MAGs tested by ANCOMBC between *G. edwardsi* and *P. lutea* are represented by black squares.

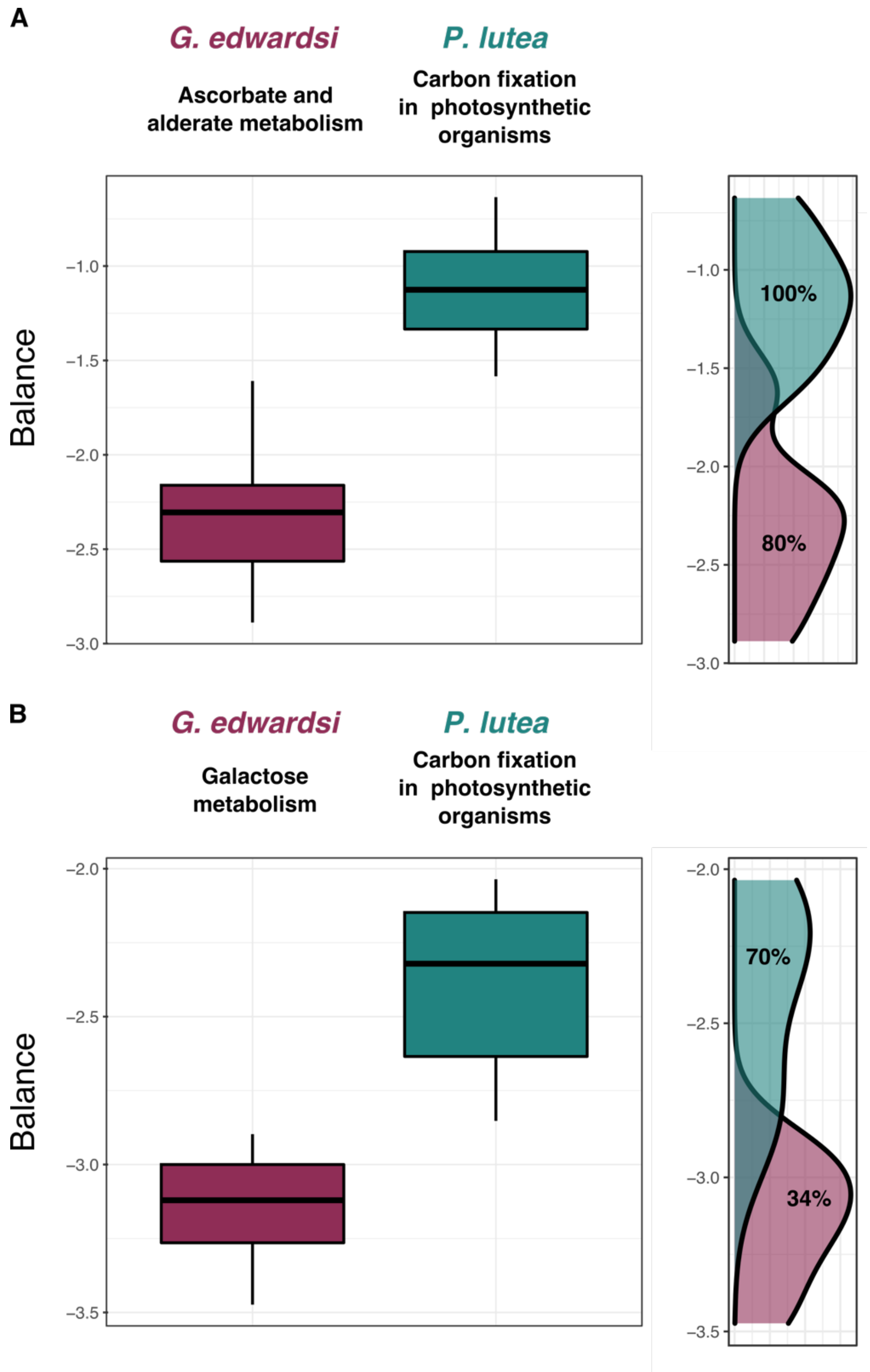


**Figure S12. Microbial functional signatures predictive of coral species.** Balances were chosen if they were included in more than 25% of cross-validation iterations and have Area Under the Curve - Receiver Operator Characteristic (AUC-ROC) > 0.8. ROC curves. Boxplots and density curves show the distribution of balance scores for each coral species, and percentages shown on the curves represent the proportion that each KEGG pathway was considered during cross-validation for **(A)** endolithic band and **(B)** skeleton metagenomes.

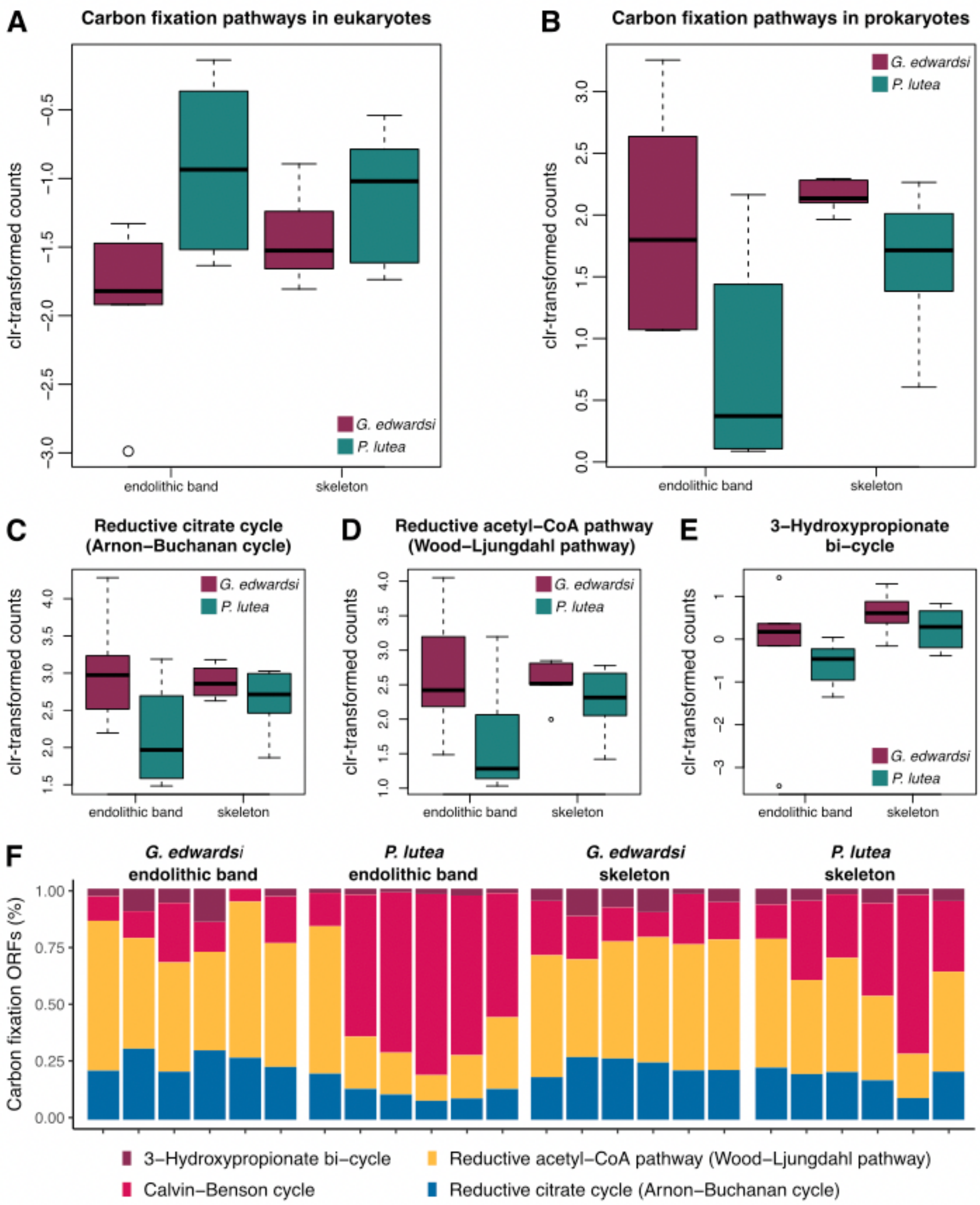


=

**Figure S13. Carbon fixation pathways in metagenomic samples.** Abundances shown as clr-transformed counts of metagenomic ORFs annotated to the L3 KEGG pathways **(A)** Carbon fixation in photosynthetic organisms (map00710), **(B)** Carbon fixation pathways in prokaryotes (ko00720), as well as the KEGG modules **(C)** reductive citric acid cycle (Arnon-Buchanan cycle) (M00173), **(D)** reductive acetyl-CoA pathway (Wood-Ljungdahl pathway) (M00377), and **(E)** 3-hydroxypropionate bicycle (M00376). **(F)** Bar plots show the relative abundance of each pathway to the total gene counts involved in carbon fixation per sample. Pathway counts were normalized by the number of KOs on each pathway.

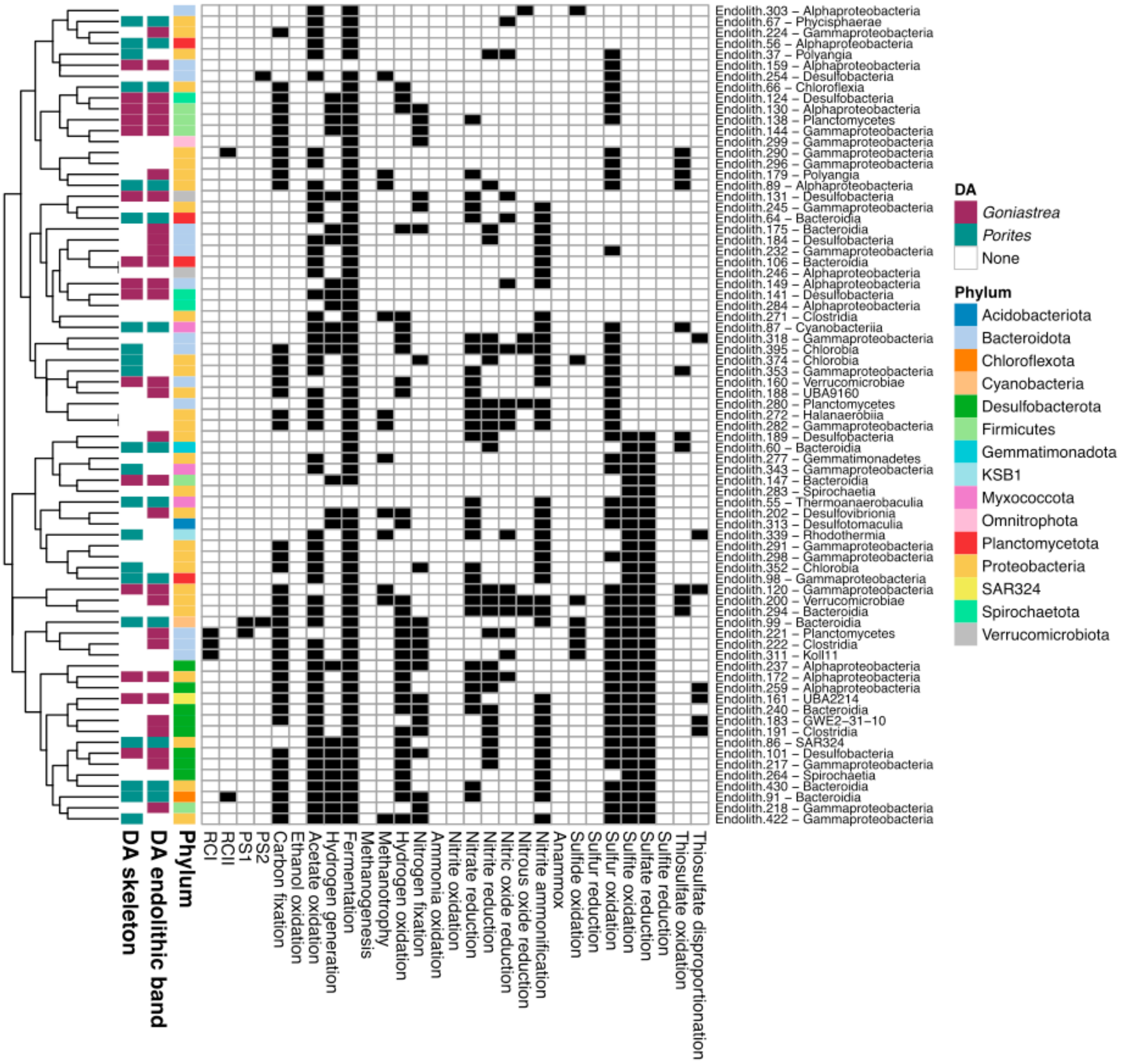


**Figure S14. Carbon, nitrogen and sulfur metabolic potential of MAGs binned from *G. edwardsi* and *P. lutea* endolithic compartments.** Differentially abundant (DA) MAGs across the different compartments tested by ANCOMBC between *G. edwardsi* and *P. lutea* are represented in the first two columns and the taxonomic affiliation of each MAG at the Phylum level is represented by colors in the third column. Presence and absence of processes involved in carbon (columns 4-14), nitrogen (columns 16-24) and sulfur (columns 25-32) cycling are denoted by black (present) and white (absent) colors.

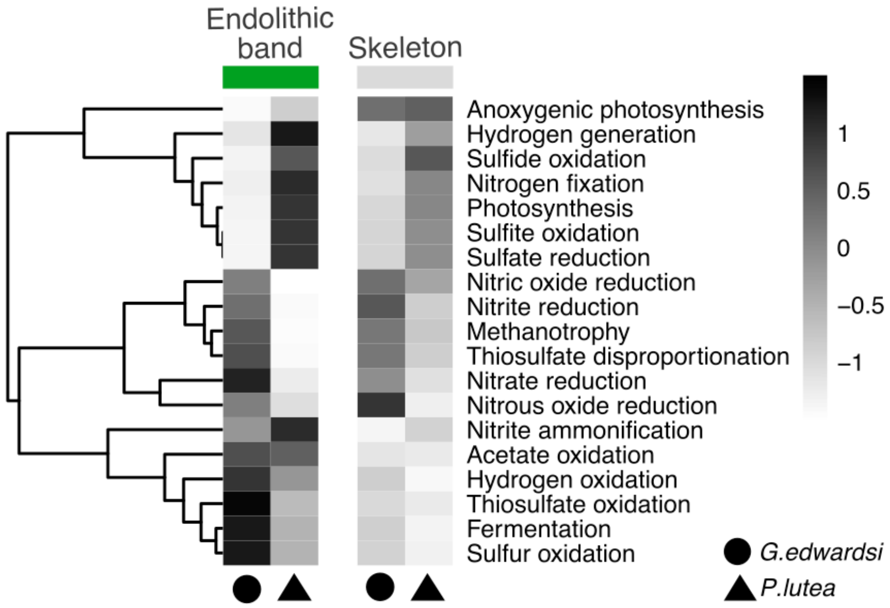


**Figure S15. Contributions of each pathway to the total binned fraction**. Z-score normalization of MAGs clr-transformed abundances was done by processes (rows) and hierarchical clustering was done using the complete method.

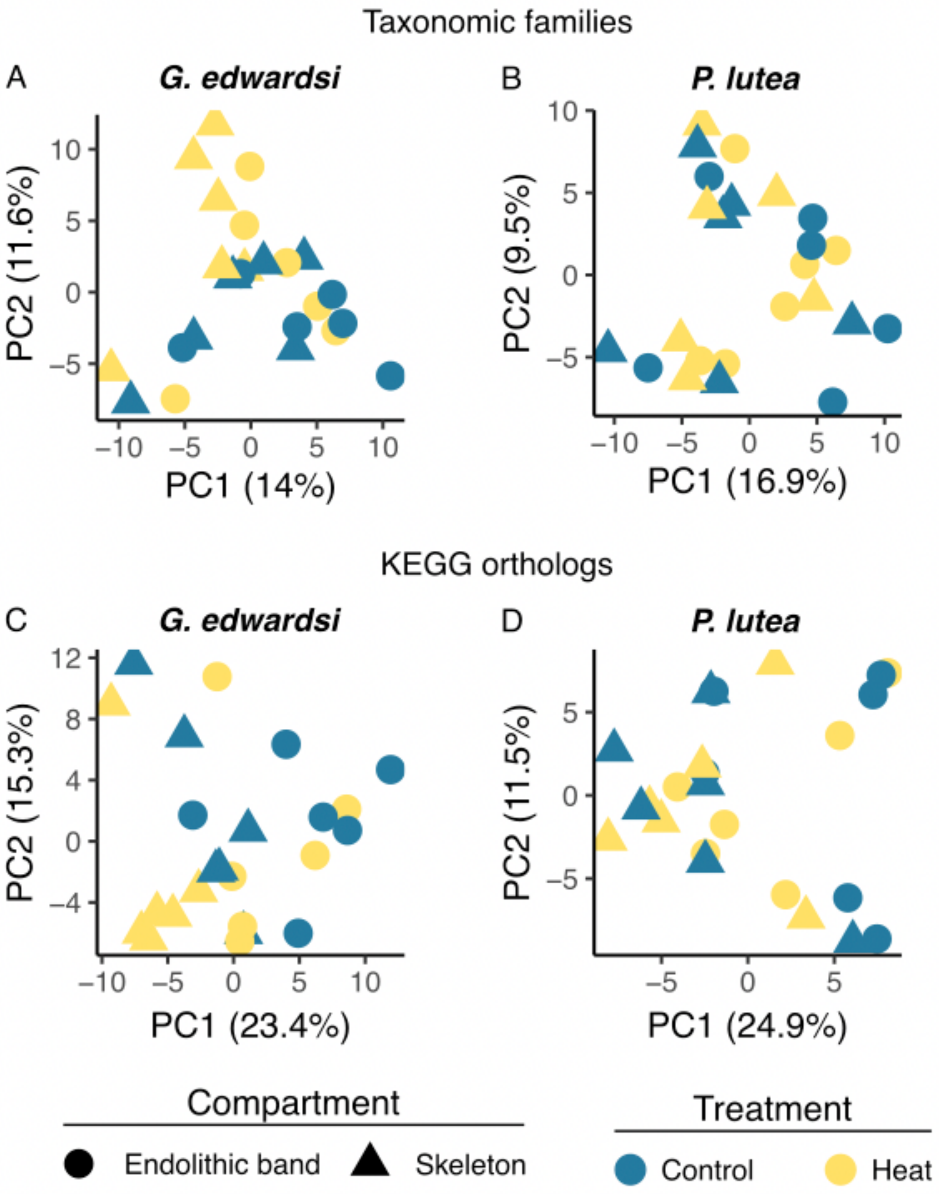


**Figure S16.** Ordination plots of microbial taxonomic and functional diversity between control and heat-stress samples.

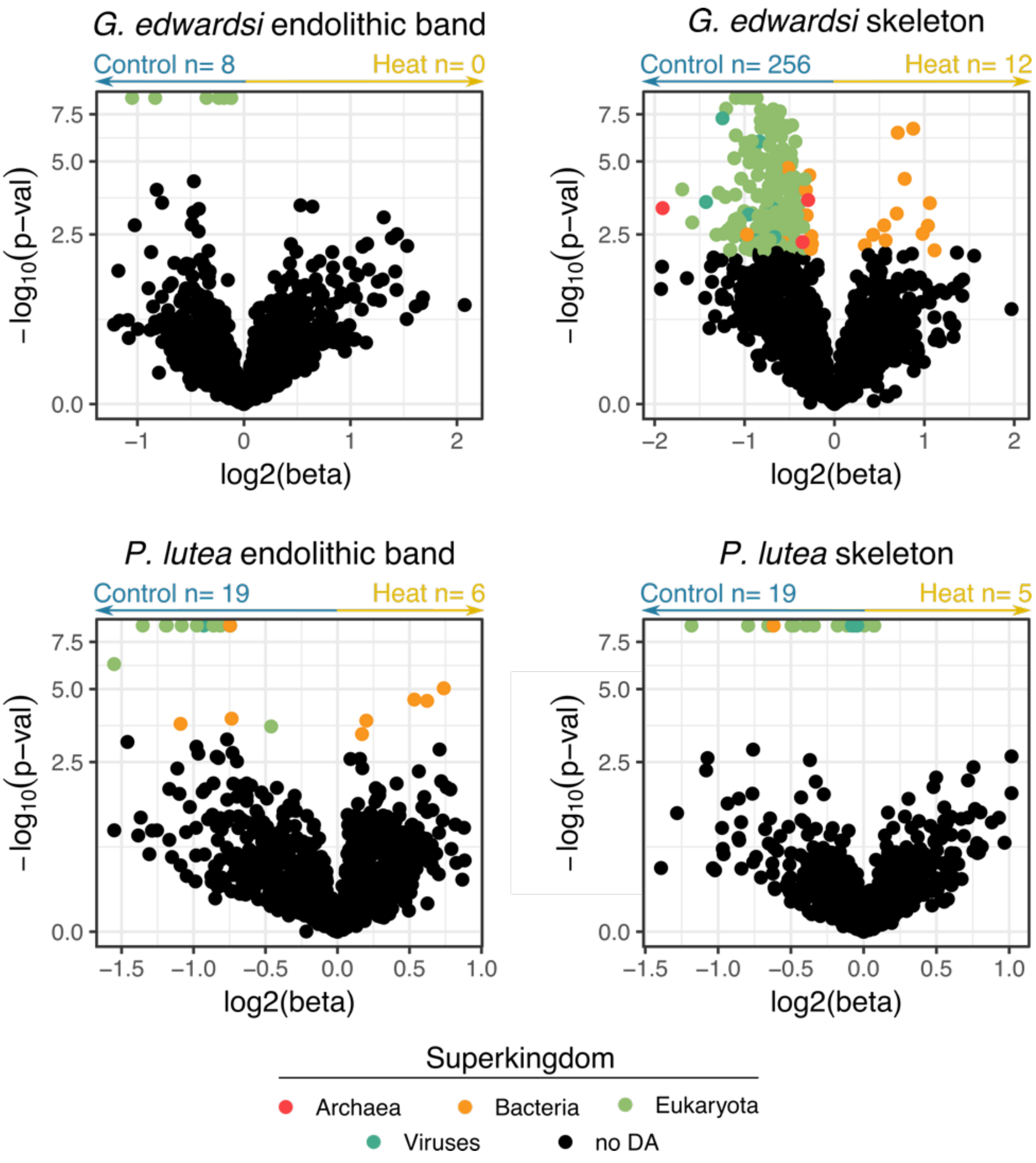


**Figure S17. Volcano plots depicting differentially abundant taxa between control and heat.** Each dot represents a taxonomical family. Significantly abundant families (fdr-adjusted p-value < 0.05) were colored by superkingdom and no differentially abundant families were colored in black. Effect sizes were represented by the $log_2$ of the Beta statistic obtained from ANCOM-BC.

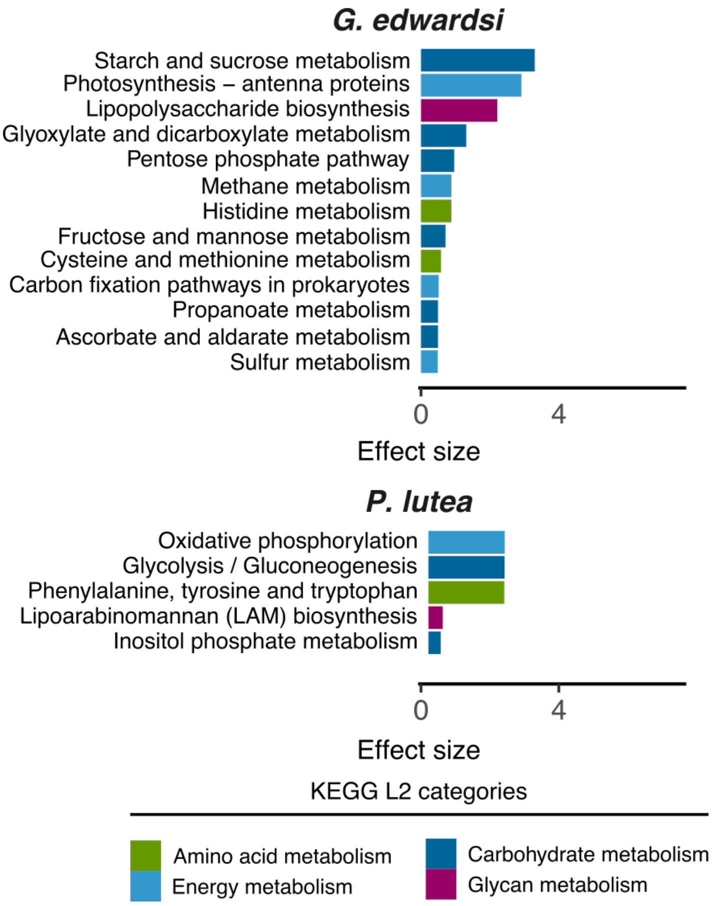


**Figure S18. Processes enriched during thermal stress in the endolithic band.** Bar plots show the effect size of L3 KEGG pathways associated to differentially abundant KOs involved in metabolism between control and heat samples in *G. edwardsi* and *P. lutea* endolithic band.

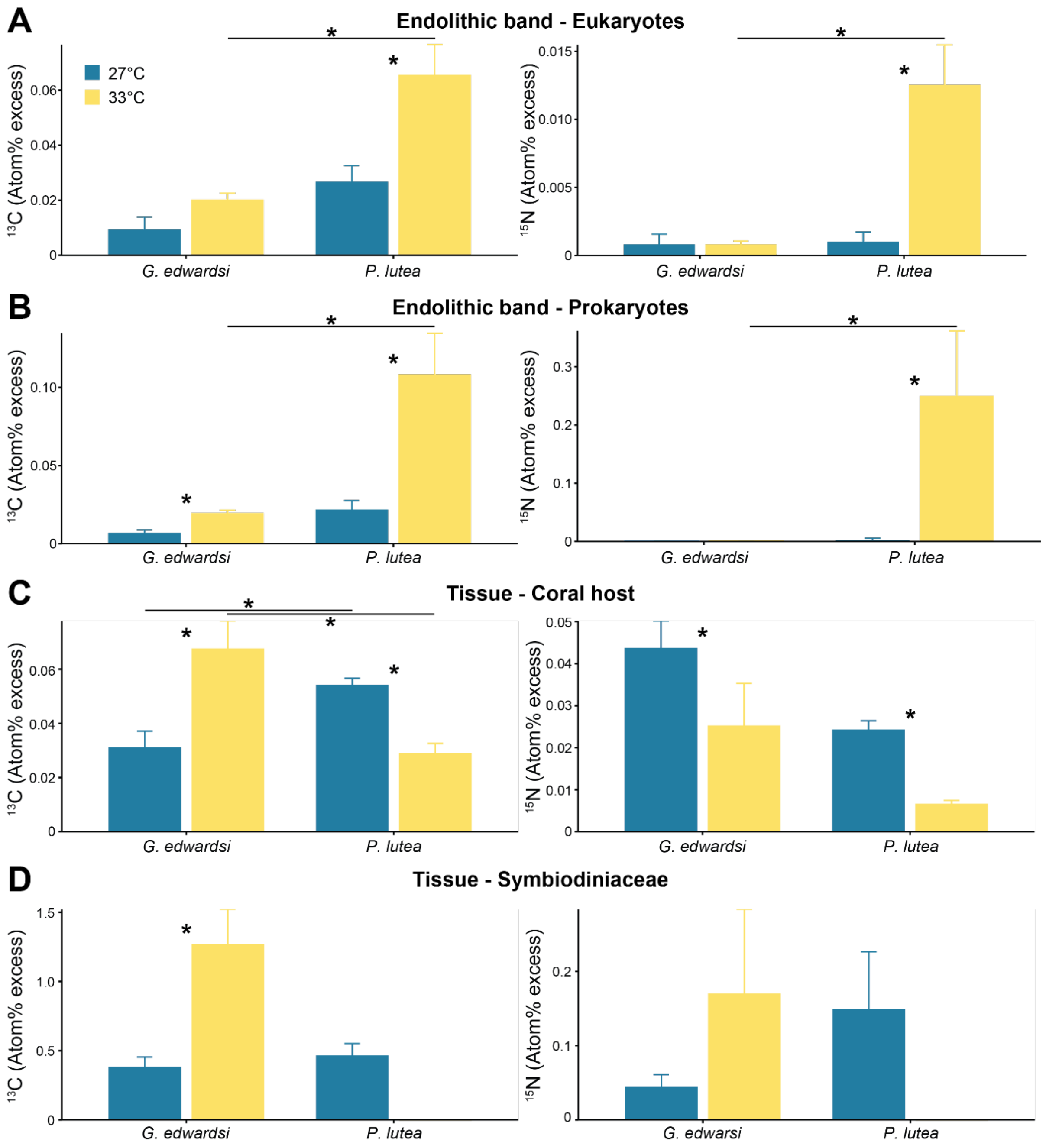


**Figure S19. Assimilation of the stable isotopes $^{13}C$ and $^{15}N$ in the tissues and endolithic band of *G. edwardsi* and *P. lutea* subjected to heat stress.** NanoSIMS quantification of stable isotope assimilation in (**A**) eukaryotes from the endolithic band (cells larger than 3 $\mu$m), **(B)** prokaryotes from the endolithic band (cells smaller than 3 $\mu$m), **(C)** coral host tissues, and (**D**) Symbiodiniaceae cells. Asterisks denote significant differences (Kruskal-Wallis test, $p < 0.01$). Detailed statistic tables can be found in Table S11.
